# Investigating the Trichosanthis Pericarpium - Trichosanthis Radix herbal pair’s role in alleviating COPD through gut microbiota function, metabolomics analysis and cell validation experiment

**DOI:** 10.1371/journal.pone.0330621

**Published:** 2025-08-22

**Authors:** Yushan Zhao, Xiaoying Tan, Tian Wang, Yuxuan Yuan, Kelei Su, Erxin Shang, Qingling Xiao, Sheng Guo, Shulan Su, Huangqin Zhang, Jin-ao Duan, Pei Liu

**Affiliations:** 1 Jiangsu Provincial Key Laboratory of Functional Substances in Traditional Chinese Medicine Formulae and Innovative Drug Discovery, Jiangsu Collaborative Innovation Center of Chinese Medicinal Resources Industrialization, National and Local Collaborative Engineering Center of Chinese Medicinal Resources Industrialization and Formulae Innovative Medicine, Nanjing University of Chinese Medicine, Nanjing, China; 2 Affiliated Hospital of Integrated Traditional Chinese and Western Medicine, Third Clinical Medical College, Nanjing University of Chinese Medicine, Nanjing, China; 3 State Key Laboratory of Technologies for Chinese Medicine Pharmaceutical Process Control and Intelligent Manufacture, Nanjing University of Chinese Medicine, Nanjing, China; Hong Kong Baptist University, HONG KONG

## Abstract

The herbal pair of Trichosanthis Pericarpium – Trichosanthis Radix (TP-TR) is derived from the classic prescription Bei-Mu-Gua-Lou-San in Yixue Xinwu, which is commonly used to treat lung heat and cough. Both originate from *Trichosanthes kirilowii* Maxim, a medicinal plant known for its effects to clear heat, dissolve phlegm, promote salivation, and relieve dryness. However, the compatibility, pharmacological synergy and gut-lung axis regulation mechanisms of TP-TR remain unclear. This study innovatively explores the therapeutic effects and underlying mechanisms of TP-TR in COPD through microbiome and amino acid metabolism. A COPD rat model was established to evaluate the efficacy of TP-TR. The gut microbiota was analyzed with 16S rRNA sequencing, while the metabolites in serum and lung were analyzed by UPLC-MS/MS. Functional prediction of the microbiome and differential metabolite analysis were performed using KEGG/SMP. The LPS, CSE – induced cell model was used to validate the impact of TP-TR and its active components on amino acid metabolism. The results demonstrated that TP-TR significantly improved pulmonary function, alleviated inflammation, modulated gut microbiota composition (e.g., *Lactobacillus*, *g_ Novosphingobium*), and regulated metabolic disturbances in COPD rats. Notably, amino acid metabolic pathways were consistently enriched across microbiota function prediction and untargeted metabolomic analyses of serum and lung. Targeted metabolomics further confirmed alterations in amino acid levels. Moreover, TP-TR, along with cucurbitacin B, cynaroside, glutamine, guanine, and apigenin induced alterations in the amino acid content of model cells. These findings reveal a novel mechanism by which TP-TR ameliorates COPD through gut microbiota regulation and amino acid metabolism modulation along the gut-lung axis.

## Introduction

Chronic obstructive pulmonary disease (COPD) is widely regarded as a respiratory disease characterized by limited airflow. The Global Strategy for the Diagnosis, Management, and Prevention of Chronic Obstructive Pulmonary Disease Report (2023) emphasizes its heterogeneous and progressive nature [1]. COPD results from a combination of genetic, environmental, and lifestyle factors throughout an individual’s lifetime [2]. As environmental damage and smoking persist, the number of patients with COPD continues to increase every year [3]. The pathological mechanisms of COPD, including the systemic inflammatory response [4], oxidative stress [5], and the imbalance of protease and antiprotease mechanisms [6] have been extensively researched. Current therapeutic agents for COPD target various pathological aspects, such as inhaled dual-action muscarinic antagonists and β2-agonists, as well as oral anti-inflammatory and antioxidant drugs [7]. However, these treatments do not fully reverse all COPD symptoms, such as dyspnea, cough, and sputum production, owing to their heterogeneous and progressive nature.

In Chinese medicine, the preferred formulas for the treatment of COPD typically include heat-clearing and phlegm-resolving herbs [8]. As the smallest compatibility unit of a famous formula “Beimu Gualou San” (Yi-Xue-Xin-Wu, Qing dynasty) for treating COPD with syndrome of phlegm and heat accumulated in lung, the herbal pair of Trichosanthis Pericarpium – Trichosanthis Radix (TP-TR) plays an important role. Trichosanthis Pericarpium (TP) contains several beneficial chemical components, particularly for acute exacerbations of COPD, especially when phlegm heat obstructs the lungs. It is commonly used to regulate qi and clear lungs. The mechanism of action may be linked to the total amino acids, nucleosides, saturated fatty acids, and other components of TP [9]. Trichosanthis Radix (TR) is rich in amino acids and polysaccharides and exhibits hypoglycemic, antitumor, anti-inflammatory, and antibacterial activities [10]. When the clinical prescription was combined with TP-TR, the effect of treating lung heat cough doubled [11]. These multi-component, multi-target mechanisms collectively highlight the therapeutic advantages of TP-TR in clinical applications and demonstrate its ability to comprehensively regulate interconnected pathological pathways, which is a key feature of the holistic approach in traditional Chinese medicine.

A deeper understanding of COPD pathogenesis requires expanding beyond pulmonary pathology to consider its systemic manifestations. Increasing evidence indicates that COPD is not merely a local lung disease but involves significant systemic alterations, among which metabolic remodeling (especially amino acid metabolism) [12] and gut microbiota dysbiosis have gained attention [13]. Chronic systemic inflammation and oxidative stress are believed to drive metabolic disturbances in COPD [14], with imbalances in specific amino acids (e.g., branched-chain amino acids and tryptophan metabolites) associated with disease severity and immune dysfunction [15]. Meanwhile, the emerging concept of the “lung-gut axis” describes the bidirectional communication between the lungs and the gut via microbiota, immune cells, and microbial metabolites [16]. In COPD, gut dysbiosis may impair intestinal barrier function, promote bacterial translocation, and exacerbate systemic inflammation [17]. Importantly, the gut microbiota plays a key role in regulating host amino acid metabolism, influencing their absorption, utilization, and transformation into bioactive metabolites (e.g., short-chain fatty acids), which can affect distant organs such as the lungs [18]. Conversely, pulmonary inflammation may also influence gut microbiota composition [19]. Thus, a cycle of “inflammation-metabolism-microbiota” may be formed in COPD. This mechanistic framework resonates with the traditional Chinese medicine theory of the “lung-large intestine interior-exterior relationship”.

Liu et al [11] previously showed that TP-TR (1:2) could modulate the composition of the gut microbiota in mice with coughing. However, the specific mechanism by which TP-TR affects COPD remains unclear. In the present study, we assessed the efficacy of TP-TR in a rat model of COPD. We analyzed the gut microbiome using 16S rRNA sequencing, performed untargeted metabolomic analysis of serum and lung tissues, and validated changes in the levels of amino acid metabolites using UPLC-MS/MS. Additionally, we validated the effect of TP-TR and its active components on amino acid metabolism using a human bronchial epithelial cell line (HBE135-E6E7) to explore the mechanism by which TP-TR intervenes in COPD.

## Materials and methods

### Samples

Trichosanthis Pericarpium (Lot No. 271220110) and Trichosanthis Radix (Lot No. 210119) were procured from Bozhou Zirui Pharmaceutical Co., Ltd. and Suzhou Tianling Chinese Medicine Decoction Pieces Co., Ltd., respectively. Professor Ling Dong from the Institute of Horticulture, Anhui Academy of Agricultural Sciences, identified them as dry mature peels and roots of *Trichosanthes kirilowii* Maxim.

According to the method described in reference [11], different ratios of TP-TR (1:0, 0:1, and 1:2) were weighed (300 g each) using the same batch of herbal materials. The extraction of the mixed herbs by heat reflux was performed following the same procedure. Specifically, the materials were extracted by refluxing with 8 volumes of purified water for 1 hour. The residue was then refluxed with 6 volumes of water for another 1 h. Subsequently, the residue was extracted twice with 8 and 6 volumes of 80% ethanol, respectively, each for 1 h. The temperature during aqueous extraction was maintained at approximately 100 °C, and at 78−80 °C during ethanol extraction. All four filtrates were collected through gauze filtration, combined, and concentrated under vacuum at 60 °C to obtain the extract (1 g/mL), which was stored at −20 °C. For the cell experiment, a portion of the TP-TR extract was concentrated under reduced pressure and lyophilized at −70 °C, 1pa for 48 h to obtain a dry powder.

### Experimental animal

All procedures in this study were approved by the Animal Experimentation Ethics Committee of Nanjing University of Chinese Medicine (approval number: 202203A043). SPF-grade male Sprague-Dawley (SD) rats, aged 6–8 weeks, were provided by Hangzhou Medical College. All animals were acclimated for one week prior to the start of the experiments and maintained under standard conditions of alternating 12-hour light and dark cycles at a constant temperature of 25 ± 2°C and 50 ± 5% relative humidity.

To minimize animal suffering, all experimental procedures were performed by professionally trained personnel. During tracheal instillation for model induction, animals were anesthetized using 2% isoflurane inhalation to ensure a pain-free state. Throughout the experiment, animals were closely monitored for signs of distress (e.g., respiratory difficulty, abnormal behavior), and humane endpoints were applied when necessary. At the end of the study, animals were anesthetized with an intraperitoneal injection of 3% pentobarbital sodium (45 mg/kg, 0.15 mL/100 g). Once deep anesthesia was confirmed by the absence of pain reflexes, blood was collected via the abdominal aorta until death. All procedures complied with institutional ethical guidelines.

### Animal grouping and drug administration

Stratified randomization using body weight as a stratification factor was performed to minimize experimental bias. SD rats (180-220g) were randomly divided into the following nine groups (n = 6): Control group, COPD model group, dexamethasone group (0.09 mg/kg), TP-TR low dose group (1.5 g/kg), TP-TR high dose group (4.5 g/kg), TP low dose group (1.5 g/kg), TP high dose group (4.5 g/kg), TR low dose group (1.5 g/kg), TR high dose group (4.5 g/kg). The low dose was selected based on the clinically equivalent dose in “Beimu Gualou San”. The dosage in the high-dose group was three times that in the low-dose group. Based on the occurrence and developmental characteristics of COPD and relevant literature reports, we selected the classical and generally recognized COPD modeling method [20]. Except for the control group, the other rats were induced with cigarette smoke combined with an intra-airway infusion of LPS solution to establish a COPD model. On days 1 and 14 of modeling, the rats were anesthetized by intraperitoneal injection of 3% pentobarbital (45 mg/kg) and intratracheal injection of 200 μL lipopolysaccharide. Smoke induction was not performed on the day of the airway infusion. From day 2 to week 12, rats were placed in a cigarette smoke generator and exposed to cigarette smoke at a rate of 20 nL/min for 40 min (Hongtashan cigarette, tar content: 11 mg). From 13 weeks to 16, each group was administered medicine before modeling by gavage, and modeling was continued during the administration period until the end of 16 weeks. The blank control and model groups were administered an equal volume of sterile 0.9% (w/v) sodium chloride solution (normal saline) by gavage.

### Lung function analysis and blood collection

After the last dose, the rats were placed in the body trace box of the EMKA noninvasive lung function test system to detect lung function parameters, such as maximum inspiratory flow, maximum expiratory flow, tidal volume, ventilatory volume per minute, and bronchoscopic tension. Each rat was analysed for 5 min, and within 2–5 min the data were collected for processing and analysis. After non-invasive pulmonary function testing, the rats were anesthetized by intraperitoneal injection of 3% sodium pentobarbital, and blood was collected from the abdominal aorta.

### Body weight and tissue weight

Individual animals were weighed once a week. At the end of the experiment, the lungs of each animal were excised and weighed.

### Histopathologic evaluation

Tissue samples from the lung were paraffin-embedded, sliced to a thickness of 4 m, and put on separate slides. The sections were stained with hematoxylin-eosin (H&E) and Masson’s trichrome stains and observed under a light microscope [20].

### Enzyme linked immunosorbent assay and biochemical analysis

Concentrations of total interleukin-6 (IL-6), interleukin-1beta (IL-1β), interleukin-17 (IL-17), tumor necrosis factor alpha (TNF-α), superoxide dismutase (SOD) and alondialdehyde (MDA) were measured for OD values using an enzyme marker and analyzed according to the manufacturer’s instructions [21].

### Reverse transcription-quantitative polymerase chain reaction (RT-qPCR)

RT-qPCR experiment employed 6 biological replicates (six animals) and 3 technical replicates (parallel testing of the same RNA sample). All experiments were conducted following the MIQE guidelines. Lung tissues were lysed with TRIzol reagent (Sigma, Germany). RNA was extracted via total RNA extraction kit (Transgen, Beijing, China). cDNA was amplified by EasyScript One-Step gDNA Removal and cDNA Synthesis SuperMix kit (Transgen, Beijing, China). According to the steps described in TransStart Top Green qPCR SuperMix (+DyeⅡ) kit, the gene transcription level expression of factors (TLR4, TGF-β, MUC5AC, MUC5B, MMP9 and MMP12) was determined by fluorescence real-time quantitative PCR instrument. GAPDH was used as the housekeeping gene for normalization. Data were analyzed and compared after 2^-ΔΔCt^ transformation. The primer sequences used for PCR analysis are shown in S1 Table in S1 File.

### Fecal 16 S rRNA sequencing

At the end of 4 weeks of drug treatment, feces from the COPD rats were obtained under sterile conditions. Total DNA was extracted according to the instructions of the E.Z.N.A.® Soil DNA Kit (Omega Bio-tek, Norcross, GA, USA). DNA integrity was detected by 1% agarose-gel electrophoresis. The genomic DNA was used as a template for PCR amplification of the V3-V4 region of the 16S rRNA genes with barcode-specific primers 338F (5’-ACTCCTACGGGAGGCAGCAG-3’) and 806R (5’-GGACTACHVGGGTWTCTAAT-3’). PCR amplification cycling conditions were as follows: initial denaturation at 95 °C for 3 min, followed by 27 cycles of denaturing at 95 °C for 30 s, annealing at 55 °C for 30 s and extension at 72 °C for 45 s, and single extension at 72 °C for 10 min, and end at 4 °C. The PE library was constructed according to the Illumina MiSeq standard operating procedures and sequenced using the MiSeq PE300 platform. Using UPARSE software (version 7.1 http://drive5.com/uparse/), an OUT table of microorganisms was generated, and their abundance, alpha diversity, beta diversity, and differential microbiota were analyzed.

### Untargeted metabolomics study [22]

Changes in lung and serum metabolites were screened using liquid chromatography-mass spectrometry (LC-MS). An ACQUITYTM UHPLC BEH C18 chromatographic column (2.1 mm × 100 mm, 1.7μm) was used, the mobile phase was 0.1% formic acid water (A) -acetonitrile (B), gradient elution conditions, 0–3 min, 5% −45% B; 3–14 min, 45% −95% B; 14–15 min, 95% B. Column temperature: 40°C. Injection volume: 2 μL. Conditions were: electrospray positive and negative ion sources (ESI^+^, ESI^-^), sheath gas flow rate of 40 arb, auxiliary gas flow rate of 10 arb, capillary temperature of 275 ºC, spray voltage of 5 kV, tube lens voltage level of 65%, capillary voltage of 35 V, scanning range of m/z 100 ~ 1000; 60000 mass resolution; collision gas high purity nitrogen.

### Targeted metabolomics study

#### Metabolite extraction in serum and lung [23].

The rat lung tissue (100 mg) was precisely weighed and homogenized in 1 mL of water using zirconium beads. The homogenization was performed three times at 60 Hz under 4°C conditions. Subsequent ultrasonic treatment was conducted in an ice-water bath for 15 min. The sample was then centrifuged at 12000 rpm for 10 minutes at 4°C, and the resulting supernatant was carefully collected. A volume of 400 μL of the supernatant was mixed with 1200 μL of acetonitrile. The mixture was vortexed and incubated on ice for 15 min. It was centrifuged again at 12000 rpm for 10 minutes at 4°C. A volume of 600 μL of the supernatant was collected and combined with 20 μL of L-2-CPA (20 μg/mL). The samples were then concentrated to dryness using a centrifuge concentrator. The dried residues were redissolved with 200 μL 50% acetonitrile and the supernatant was filtered with 0.22 μm filter membrane for subsequent analysis.

For rat serum, 400 μL of serum was mixed with 1200 μL of acetonitrile. The mixture was vortexed and incubated on ice for 15 min. The mixture was centrifuged at 12000 rpm for 10 min at 4°C. A volume of 600 μL of the supernatant was collected and supplemented with 20 μL of L-2-CPA (20 μg/mL). The samples were then concentrated to dryness. The dried residues were redissolved with 100 μL 50% acetonitrile and the supernatant was collected for LC-MS.

#### Quantification of amino acids by LC-MS/ MS analysis.

Changes in amino acids in rat serum and lung tissues were quantitatively detected using LC-MS/MS [22], and the detailed steps were shown in S2 Table in S1 File.

### Cell experiments verified the amino acid pathway enriched in gut microbiota function and non-target metabolomes analysis

#### Drugs preparation.

Cigarette smoke extract (CSE) was prepared as previously described [24]. Briefly, two cigarettes (Hongtashan cigarette, tar content: 11 mg) without a filter were burned, and the smoke was bubbled through 5 mL of Keratinocyte Medium (KM). The pH of the suspension was adjusted to 7.4, and it was filtered through a 0.22 μm membrane. Quality was controlled by measuring the absorbance at 320 and 540 nm. If the difference was between 0.9 and 1.2, the CSE was deemed qualified and regarded as a 100% concentration of CSE. Use within one hour of dilution with the KM culture medium. LPS stock solution was prepared by dissolving 1 mg of LPS in 1 mL PBS, filtering through a 0.22 μm membrane, and diluting as needed.

Our previous study analyzed the chemical components of the TP-TR herb pair. Components included rutin (26.26 μg/g), luteolin-7-O-glucoside (5.12 μg/g), cucurbitacin B (450.97 μg/g), inosine (156.76 μg/g), L-serine (31.19 μg/g), glutamine (111.33 μg/g), guanine (54.29 μg/g), and others. The complete data can be found in S3 Table in S1 File and the original literature source [10]. Correlation analysis was conducted using SPSS Statistics 22.0, to explore the relationships between the identified differential bacterial genera, differential metabolites, and chemical components in TP-TR. Several chemical components (rutin, cynaroside, cucurbitacin B, inosine, L-serine, glutamine, guanine, and apigenin) were screened, which were highly correlated with gut microbial metabolites, and the lung and serum metabolites.

To further investigate the effects of these chemical components on amino acid metabolism in vitro, the corresponding standard substances were accurately weighed and dissolved in dimethyl sulfoxide (DMSO) to prepare stock solutions. These were further diluted with KM medium to obtain the desired working concentrations, ensuring that the final DMSO concentration in the culture medium did not exceed 0.1%. The TP-TR lyophilized powder was directly dissolved in complete KM medium to prepare a stock solution at a concentration of 1 g/mL. This stock was then diluted with complete KM medium to obtain working concentrations of 0.1, 0.5, 1, 5, 10, 25, 50, and 100 μg/mL. All dilutions were filtered through 0.22 μm microporous membranes before use to ensure sterility.

#### Cell culture and treatments.

HBE135-E6E7 cells (iCell Bioscience Inc, Shanghai) were cultured in KM basic medium (Sciencell, USA) supplemented with Keratinocyte Growth Supplement (KGS), 100-U/mL double antibiotics (Sciencell, USA) at 37°C in the atmosphere with 5% CO_2_. Cells were treated with CSE (5%) and LPS (10 μg/mL, yuanye, shanghai) to construct COPD model [25].

Cells were treated with different concentrations of “TP-TR” and chemical constituents to screen the safe concentration. The effects on the survival rate of HBE135-E6E7 cells were detected using the MTT assay. Subsequently, cells were inoculated in T75 bottle, co-cultured LPS, CSE and each group of drugs (L、M、H three doses) in 5% CO_2_ cell incubator at 37°C for 24 h. Washed three times with pre-cooled PBS, centrifuged at 1000 rpm for 5 min after trypsin digestion, resuspended with PBS, and 10^7^ cells were centrifuged again to remove the supernatant. The cell precipitate was quickly placed in liquid nitrogen for 30 s, and stored at −80°C for use.

#### Metabolite extraction in cell [26].

The samples were accurately drawn in 2 mL centrifuge tube, 300 μL 10% formic acid methanol solution and 10 μL of L-2-CPA (20 μg/mL) internal standard sample were added, along with 100 mg of glass beads, liquid nitrogen was rapidly frozen for 5 min. After thawing to room temperature, the solution was oscillated at 60 Hz for 2 min in a grinder. After centrifugation at 12000 rpm, 4°C for 5 min, 200 μL of supernatant was taken, and redissolved after centrifugal concentration. The vortex was oscillated for 30 s, and the supernatant was filtered with 0.22 μm filter membrane in the detection bottle.

#### Quantification of amino acids by LC-MS/ MS analysis.

Changes in amino acids in the cells were quantitatively detected by LC-MS/MS, as described in Section 2.11.2.

### Statistical analysis

All statistical analyses were performed using GraphPad Prism 8.0 (GraphPad Software, San Diego, CA, USA). Data are expressed as mean ± standard error of the mean (SEM). The Shapiro-Wilk test was used to evaluate the normality of data distribution, and Levene’s test was used to assess homogeneity of variances. When data met the assumptions of normality and equal variance, one-way ANOVA was performed followed by Tukey’s HSD post hoc test. If the assumption of homogeneity was violated, Dunnett’s T3 test was used. For non-normally distributed data, the Kruskal-Wallis test was applied, followed by Dunn’s multiple comparisons test. A p-value < 0.05 was considered statistically significant. To assess the strength of the experimental effects, we calculated the effect size (Cohen’s d) and provided the 95% confidence interval (CI) to quantify the statistical significance of the results (S4 Table in S1 File). LC-MS/MS data were analyzed using Analyst 1.6.3. software.

## Results

### TP-TR can improve lung function in COPD rats

**Fig 1:**
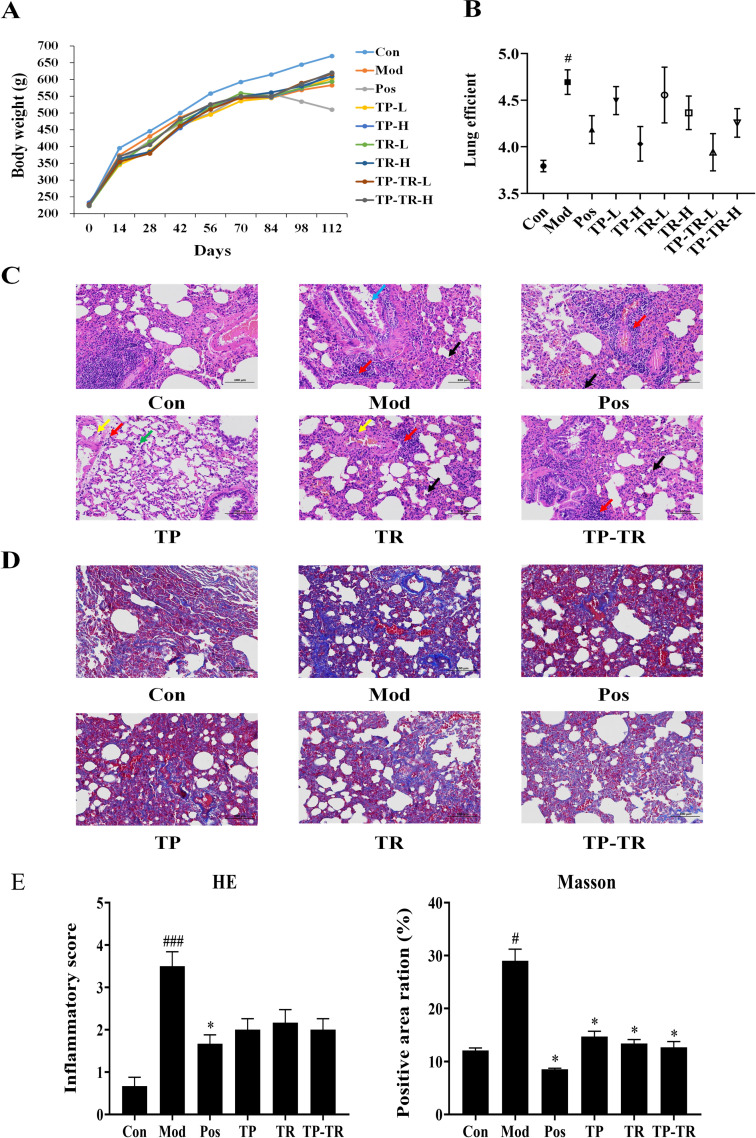
Effects of TP-TR on body weight and lung pathology in COPD rats. **(A)** Changes in body weight of rat in the COPD model. **(B)** Coefficient of lung of COPD rats. **(C)** HE pathological section of lung tissue. **(D)** Masson pathological section of lung tissue. **(E)** HE inflammation score and Masson positive area ration. Scale bar = 100 μm. Data are presented as means ± SEM, n = 6. ^#^*P* < 0.05, ^##^*P* < 0.01, ^###^*P* < 0.001, Mod VS Con; *P < 0.05, **P < 0.01, ***P < 0.001, Administration VS Mod. One-way ANOVA. (Con, normal group; Mod, model group; Pos, positive drug group; TP-L, Trichosanthis Pericarpium low dose group; TP-H, Trichosanthis Pericarpium high dose group; TR-L, Trichosanthis Radix low dose group; TR-H, Trichosanthis Radix high dose group; TP-TR-L, Herbal pair low dose group; TP-TR-H, Herbal pair low dose group.).

During the experimental period, rats in the blank control group exhibited normal diet and activity, with rapid weight gain. In contrast, rats in the COPD model group showed slow weight gain (Fig 1A), reduced food and water intake, decreased activity levels, and diminished responsiveness to external stimuli. These symptoms were alleviated after treatment. COPD rats were treated with TP-TR for four weeks to investigate the therapeutic effect of TP-TR on COPD. The lung coefficient of COPD rats was significantly higher than that of the normal control group. After administration, the lung coefficient of each group returned to 3.79 in the control group and increased to 4.69 after modeling. After the administration of TP in the low- and high-dose groups, TR in the low- and high-dose groups, and TP-TR in the low- and high-dose groups, the lung coefficients decreased to 4.50, 4.03, 4.56, 4.36, 3.94 and 4.26, respectively (Fig 1B). We scored inflammation around small airways and blood vessels. A score of “0” indicates no or very few inflammatory cells around the small airways or blood vessels. “1” indicates scattered inflammatory cell infiltration around the small airways or blood vessels. “2” indicates 1–2 layers of inflammatory cell infiltration around the small airways or blood vessels. “3” indicates 3–5 layers of inflammatory cell infiltration around the small airways or blood vessels. “4” indicates more than five layers of inflammatory cell infiltration around the small airways or blood vessels. In the lung tissue sections (Figs 1C and 1E), compared with the blank group, the alveolar wall of rats in the model group was significantly thickened in large areas, accompanied by a large amount of inflammatory cell infiltration (black arrow), uneven vascular wall thickness, small focal bleeding at the tissue edge, exfoliated epithelial cells in the lumen (blue arrow), and focal inflammatory cell infiltration around blood vessels (red arrow) were seen in the lung tissue. After the intervention, different degrees of improvement were observed in each group. In the TP group, scattered perivascular inflammatory cell infiltration and occasional focal infiltrates were observed (red arrows), along with numerous macrophages in the alveolar spaces (green arrows). Alveolar walls showed no obvious thickening, but vascular walls appeared uneven in thickness (yellow arrows), and bronchi were largely normal. In the TR group, focal inflammatory infiltration was occasionally seen around bronchi and vessels (red arrows), with marked alveolar wall thickening and extensive inflammatory cell infiltration (black arrows). Vascular walls were irregular in thickness (yellow arrows). In the TP-TR group, mild thickening of large areas of alveolar walls was observed, accompanied by widespread inflammatory cell infiltration (black arrows) and limited peribronchial focal infiltration (red arrows). The Masson staining results are shown in Figs 1D–1E. Compared to blank group, fibrous tissue and collagen fibers were significantly increased in lung tissue of rats in the model group, and the positive area ratio reached 29.02%. Compared with model group, blue collagen fibers and collagen deposition could be reduced in each administration group to varying degrees, and the positive area ratios of TP, TR, and TP-TR groups were reduced to 14.72%, 13.40%, and 12.65%, respectively.

**Fig 2:**
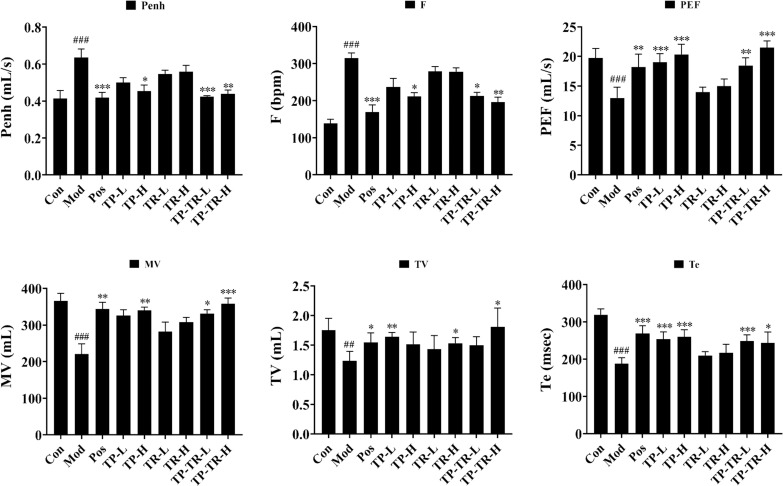
Effect of Trichosanthis Pericarpium – Trichosanthis Radix herbal pair on lung function of COPD model rats. Data are presented as means ± SEM, n = 6. ^#^*P* < 0.05, ^##^*P* < 0.01, ^###^*P* < 0.001, Mod VS Con; *P < 0.05, **P < 0.01, ***P < 0.001, Administration VS Mod.

Lung function was assessed using a non-invasive small animal lung function detector. Among the measured parameters, Penh reflects changes in airway resistance, PEF indicates the degree of airway patency, and MV and TV represent the amount of gas exchange between the lungs and the external environment per unit of time. The parameter f denotes respiratory frequency. Te reflects the rhythm and efficiency of the ventilation. Lung function test results (Fig 2) showed that, compared with the blank group, the bronchosystolic tension (*P* = 0.0002) and respiratory rate (*P* < 0.0001) of rats in the model group were significantly increased, and the tidal volume (*P* = 0.0041), maximum expiratory flow (*P* = 0.0003), volume per minute (*P* < 0.0001) and expiratory duration (*P* < 0.0001) were significantly decreased. The results showed that lung function decreased, and airway resistance increased in rats treated by cigarette smoke combined with LPS. After the intervention, the bronchoconstrictive parameters and respiratory frequency of COPD rats were significantly reduced in the TP-TR group; the maximum exhalation flow rate, minute volume, tidal volume, and exhalation duration were increased, and the effect was better in the high-dose group.

### Results of ELISA and RT-qPCR

**Fig 3:**
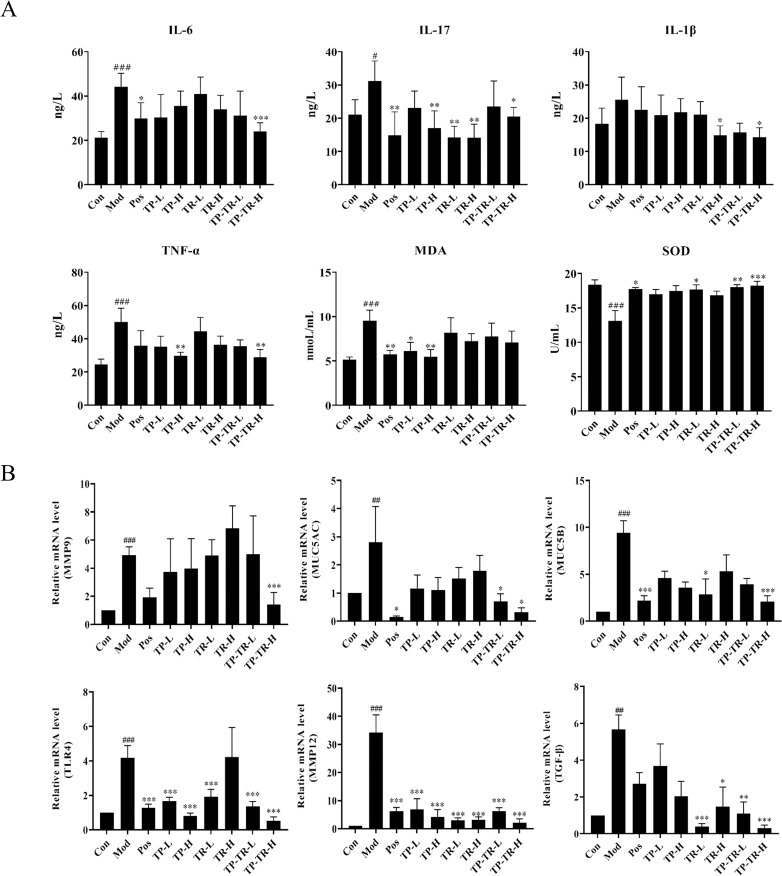
The effect of TP-TR on inflammatory factors, oxidative stress, and lung tissue-related factors in COPD rats. **(A)** Effects of different administration groups on serum inflammatory factors and oxidative stress indexes of COPD model rats. **(B)** Pulmonary tissue related factors of COPD model rats were detected in each administration group. Data are presented as means ± SEM, n = 6. ^#^*P* < 0.05, ^##^*P* < 0.01, ^###^*P* < 0.001, Mod VS Con; *P < 0.05, **P < 0.01, ***P < 0.001, Administration VS Mod.

The detection results of serum inflammatory factors of rats in each group showed (Fig 3A) that compared with the blank group, the serum levels of IL-6 (*P* = 0.0004), IL-17 (*P* = 0.0454) and TNF-α (*P* < 0.0001) of rats in the model group were significantly increased. Compared with the model group, the release of inflammatory factors in rats in each administration group was reduced to varying degrees, among which the TP-TR-H group had the best effect. The results of the oxidative stress index showed that, compared with the blank group, the SOD content in the model group was significantly decreased (*P* < 0.0001) and the MDA content was significantly increased (*P* < 0.0001). Compared with the model group, the SOD and MDA levels in each administration group could be adjusted to different degrees to improve the oxidative stress state, among which the TP-TR-H group had the best effect.

The RT-qPCR results of qPCR detection were shown in Fig 3B. Compared with blank group, TLR4, TGF-β, MUC5AC, MUC5B, MMP9 and MMP12 in the model group were significantly increased. Compared to the model group, after the drug administration intervention, all relevant factors in each group could be adjusted back to different degrees. In conclusion, TP-TR can reduce the expression of TLR4 and TGF-β in COPD rats, thus alleviating airway inflammation and pulmonary fibrosis, reducing the expression of MUC5AC and MUC5B, alleviating airway mucus hypersecretion, and reducing the expression of MMP9 and MMP12 to delay lung tissue destruction and improve lung function.

Immunofluorescence analysis provided support for the RT-qPCR results, demonstrating that TP-TR significantly reduced the expression levels of MMP9, TLR4, and TGF-β in lung tissues of COPD rats. Detailed experimental methods are documented in Supplementary material (S1 Text in S1 File), with corresponding experimental results presented in S1 Fig.

### TP-TR alleviates smoke-induced gut microbiota disorder in COPD rats

**Fig 4:**
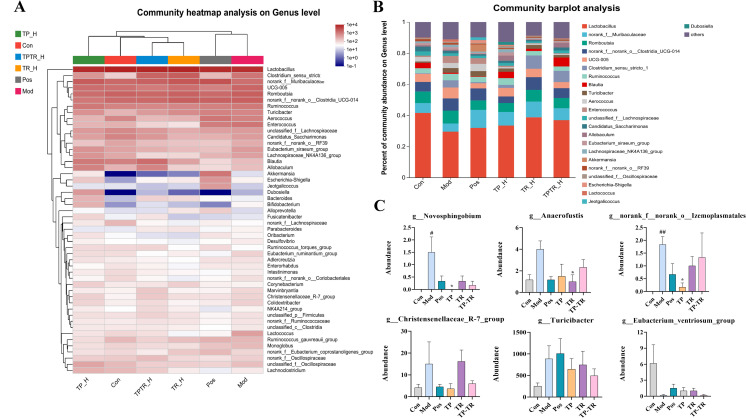
Composition and analysis of gut microbiota in COPD model rats. **(A)** The heatmap of prevention and model period in genus level. **(B)** Microbial community bar plot by genus. (Composition analysis of gut microbiota in rat colitis). **(C)** Difference analysis of gut microbiota based on genus level. Data are presented as means ± SEM, n = 6. ^#^*P* < 0.05, ^##^*P* < 0.01, Mod VS Con; *P < 0.05, Administration VS Mod.

Microbial sequencing was performed on the intestinal contents of rats with COPD treated with cigarette smoke. The results of the dilution curve and species composition showed that the sample species for sequencing in this experiment were abundant and highly uniform and that the amount of sequencing data was reasonable (S2A–2C Figs). Alpha diversity reflects the species diversity within a specific area. Compared to the normal group, the model group showed an increase in the Ace, Chao, and Shannon indices, while the Simpson index decreased. Following treatment, the indices of each group are adjusted back towards the levels of the normal group (S2D–2G Figs). The results showed that the gut microbiota of COPD rats was disturbed and the microbial community changed after smoke exposure (Fig 4A–4B). Compared to the blank group, the abundances of *Lactobacillus*, *norank_f_Muribaculaceae*, *Ruminococcus_torques_group*, *Blautia* and other genera were downregulated in the model group. The abundances of *Romboutsia*, *Zurich bacillus genus turicibacter*, *Enterococcus*, *unclassified_f_Oscillospiraceae*, *norank_f_norank_o_Clostridia_UCG - 014*, *UCG −005*, *Clostridium_sensu_stricto_1*, *Ruminococcus*, and others were upregulated. Compared to the model group, the abundance of *Lactobacillus*, *norank_f_Muribaculaceae*, *Blautia*, *Allobaculum* and other bacteria in the TP group was upregulated after drug intervention. The abundance of *Romboutsia*, *norank_f_norank_o_Clostridia_UCG-014*, *UCG-005*, *Clostridium_sensu_stricto_1, turicibacter*, and *Aerococcus* was lower. The abundance of *Lactobacillus*, *norank_f_Muribaculaceae*, *norank_f_norank_o_Clostridia_UCG-014* and *Clostridium_sensu_stricto_1* bacteria was upregulated in the TR group and decreased the abundance of *UCG-005*, *Ruminococcus*, *Allobaculum*, etc. The abundance of *Lactobacillus*, *norank_f_Muribaculaceae*, *Clostridium_sensu_stricto_1*, *Blautia* and other bacterial genera was upregulated in the TP-TR group. The abundances of *Romboutsia*, *norank_f_norank_o_Clostridia_UCG-014*, *UCG-005*, *Ruminococcus*, *turicibacter*, *Aerococcus* and other genera decreased.

### Analysis of different bacteria and drug regulation

LEfSe analysis results showed (S3 Fig) that a total of 13 different bacterial genera were identified between the blank and model group. *Methylobacterium*, *Eubacterium_ventriosum*, *Clostridium_sensu_stricto_1*, *Turicibacter*, *Izemoplasmatales*, *Christensenellaceae_ R-7_group*, *Anaerofustis*, *Novosphingobium*, *Escherichia-Shigella*, *Akkermansia*, *Bacteroides*. *G_methylobacter-methylorubrum* was most abundant in the blank group, and *g_Clostridium_sensu_stricto_1* had the highest abundance in the model group. The statistical results of the multigroup difference test showed that the abundance of *g_Novosphingobium and g_norank_f_norank_o_Izemoplasmatales* in COPD rats increased significantly, and this abundance was reversed after administration. The abundance of *g_Novosphingobium* in the blank group was 0% and increased to 1.5% in the model group. After administration, the abundance of *g_Novosphingobium* in the TP, TR, and TP-TR groups decreased to 0%, 0.33%, 0.17%, respectively. The relative abundance of *g_Anaerofustis* in the blank group was 1.17%, and increased to 4.00% in the model group. After intervention, the abundance of *G_anaerofustis* in TP, TR, and TP-TR groups decreased by 1.50%, 1.00%, and 2.33%, respectively. The abundance of *g_norank_f_norank_o_Izemoplasmatales* in blank group was 0% and increased to 1.83% in the model group. After administration, the abundance of *G_norank_o_izemoplasmatales* in the TP, TR, and TP-TR groups decreased by 0.17%. 1.00% and 1.33% (Fig 4C).

The functions of the gut microbiota were predicted using the PICRUSt2 software, which analyzed based on OTU representative sequences and associates them with the KEGG database (KEGG: https://www.genome.jp/kegg/). PICRUSt2 includes several tools: HMMER for aligning OTU representative sequences with reference sequences, EPA-NG and Gappa for embedding these sequences into a reference tree, the castor was used to normalize the 16S gene copies and MinPath for predicting gene families and mapping them to relevant metabolic pathways. The entire analysis followed the standard PICRUSt2 protocol. By comparing with the gene functions in the KEGG database, we predicted the metabolic pathways and functional modules of the gut microbiota in the COPD model (S5 Table in S1 File, S4 Fig). These findings indicated that the first-level functional layer encompasses metabolism, cellular processes, organismal systems, and genetic information processing. Further analysis of the secondary functional layer revealed that the primary functions included amino acid metabolism and biosynthesis of secondary metabolites. These metabolic pathways may affect the progression and symptoms of COPD by regulating inflammatory responses or oxidative stress.

### Metabolomics analysis

**Fig 5:**
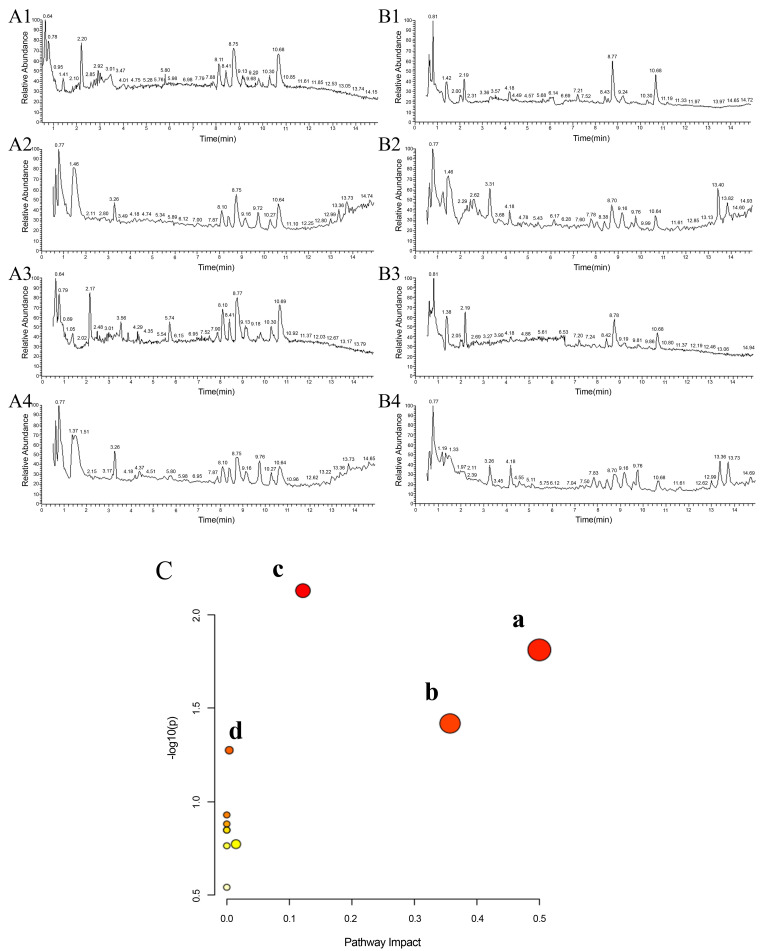
Analysis of metabolites and metabolic pathways in serum and lung tissue. **(A)** Total ion chromatogram of serum (control group, A1 positive, A2 negative; model group, A3 positive, A4 negative); **(B)** Total ion chromatogram of lung tissue (control group, B1 positive, B2 negative; model group, B3 positive, B4 negative). **(C)** Summary of pathway analysis of potential metabolites. (a. Phenylalanine, tyrosine and tryptophan biosynthesis; **b.** Phenylalanine metabolism; **c.** Glycerophospholipid metabolism; **d.** Fatty acid biosynthesis).

**Fig 6:**
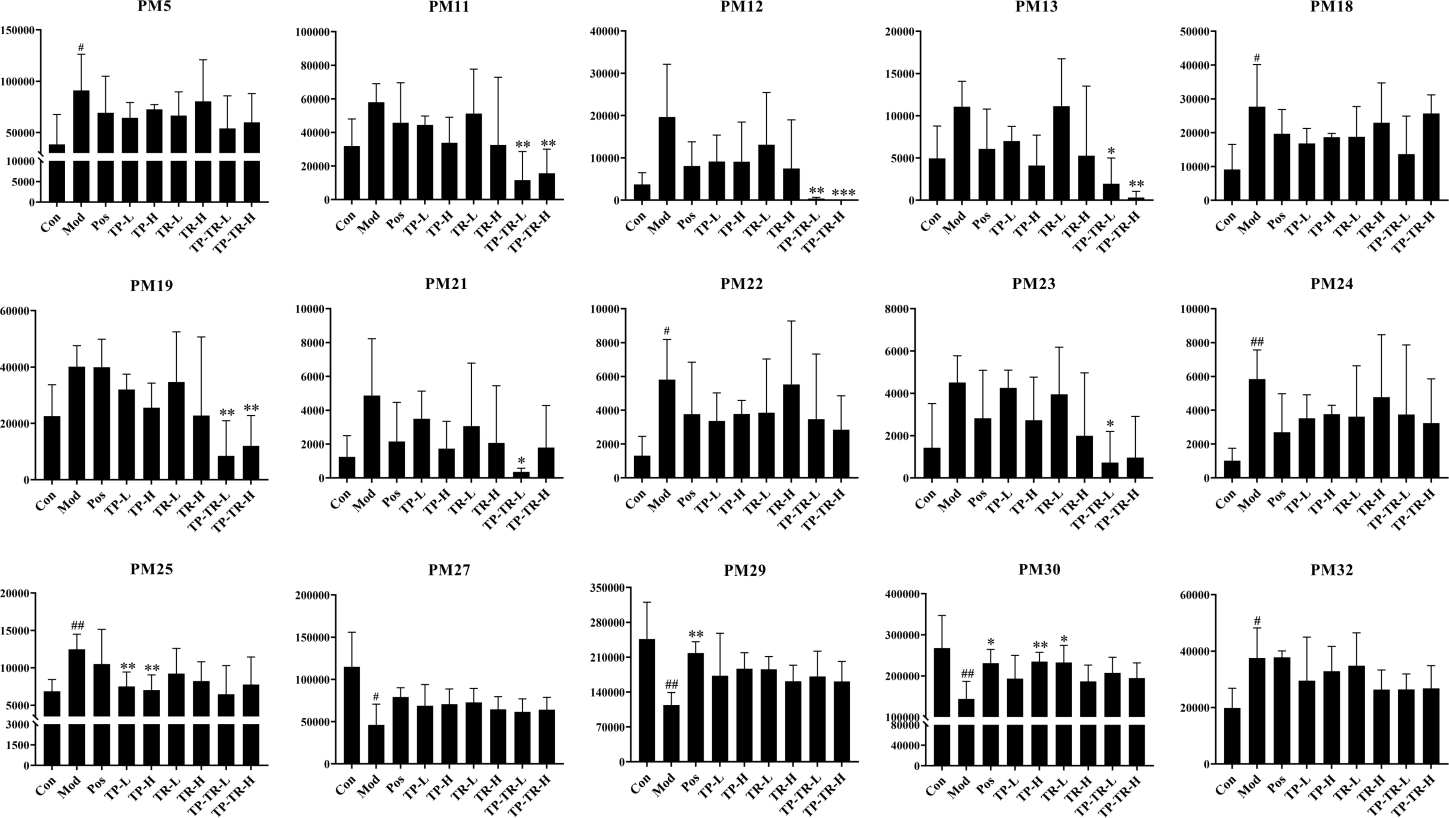
Relative peak areas of potential markers in serum and lung tissue in positive and negative ion mode. Data are presented as means ± SEM, n = 6. ^#^*P* < 0.05, ^##^*P* < 0.001, ^###^*P* < 0.001, Mod VS Con; *P < 0.05, **P < 0.01, ***P < 0.001, Administration VS Mod.

The results of the total ion flow diagram (Fig 5A–5B) and OPLS-DA (S5 Fig) analyses showed that metabolites in the serum and lung tissue samples of COPD rats were significantly different between the blank control group and COPD rats, indicating obvious metabolic abnormalities in COPD rats induced by LPS and smoke. Using a VIP value > 1, | S-Plot | > 0.05 screen potential biomarkers in the serum and lung tissue of the COPD rats and obtained 33 different metabolites (S6 Table in S1 File). The regulatory effects of drug administration on the different metabolites in each group are shown in Fig 6 S1 File (S6 Fig). Subsequently, the metabolic pathways involving these 33 differential metabolites were analyzed (Fig 5C, S7 Table in S1 File), and the results showed that COPD mainly affected the biosynthesis of phenylalanine, tyrosine, and tryptophan (influence value = 0.50), phenylalanine metabolism (influence value = 0.36), and glycinate metabolism (influence value = 0.12). It also influences fatty acid biosynthesis. The biosynthetic pathways of phenylalanine, tyrosine, and tryptophan had the highest influence, indicating that this pathway is important for the intervention of COPD and suggesting that TP-TR can intervene in the occurrence and development of COPD by regulating the biosynthesis of phenylalanine, tyrosine, and tryptophan.

### Analysis of amino acids content in rat serum and lung tissue

**Fig 7:**
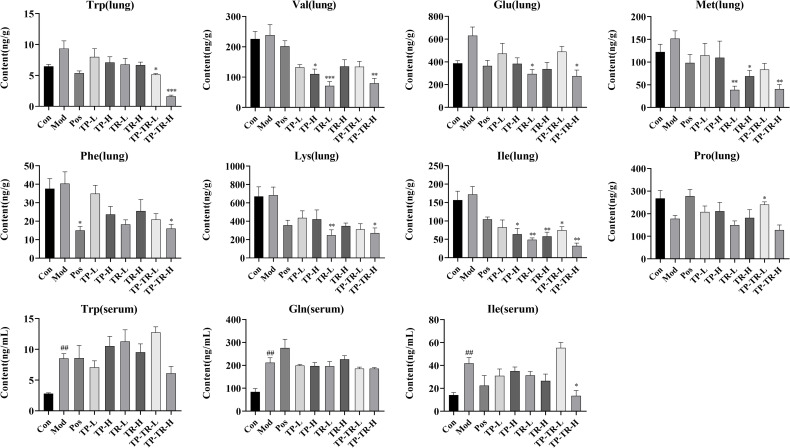
Determination results of amino acid components in rat serum and lung tissue. Data are presented as means ± SEM, n = 6. ^##^*P* < 0.01, Mod VS Con; *P < 0.05, **P < 0.01, ***P < 0.001, Administration VS Mod.

We analyzed the levels of 17 different amino acids in the rat serum and lung tissue. The results revealed the following changes (Fig 7, S7 Fig): compared to the blank group, the model group exhibited increases in tryptophan (serum and lung), glutamic acid (lung), methionine (lung), phenylalanine (lung), isoleucine (serum) and glutamine (serum) levels, whereas proline (lung) levels decreased. After drug administration, TP treatment showed a significant callback in valine and isoleucine levels. The valine, glutamic acid, methionine, lysine and isoleucine levels exhibited a significant callback in the TR group. The levels of tryptophan, valine, glutamic acid, methionine, phenylalanine, lysine, isoleucine, proline exhibited a significant callback in the TP-TR group.

The correlation analysis results for the amino acid content, gut microbiota abundance, and chemical components of TP-TR are shown in S8 Fig. The key findings included that *g_norank_f_norank_o_Izemoplasmatales* were significantly correlated with proline and threonine levels. *G_Christensenellaceae_R-7_group* showed a significant correlation with phenylalanine, whereas *g_Anaerofustis* was significantly correlated with valine. *G_Eubacterium_ventriosum_group* exhibited significant correlations with tryptophan, methionine, and glutamine levels. *G_Novosphingobium* significantly correlated with leucine levels, whereas *g_Turicibacter* correlated with leucine and serine levels. Moreover, among the chemical components of TP-TR, those associated with different gut microbiota, such as cynaroside, apigenin, glutamine, luteolin, threonine and serine, were significantly correlated with amino acid metabolites.

### Analysis of amino acids content by LC-MS/ MS in HBE135-E6E7 cells

**Fig 8:**
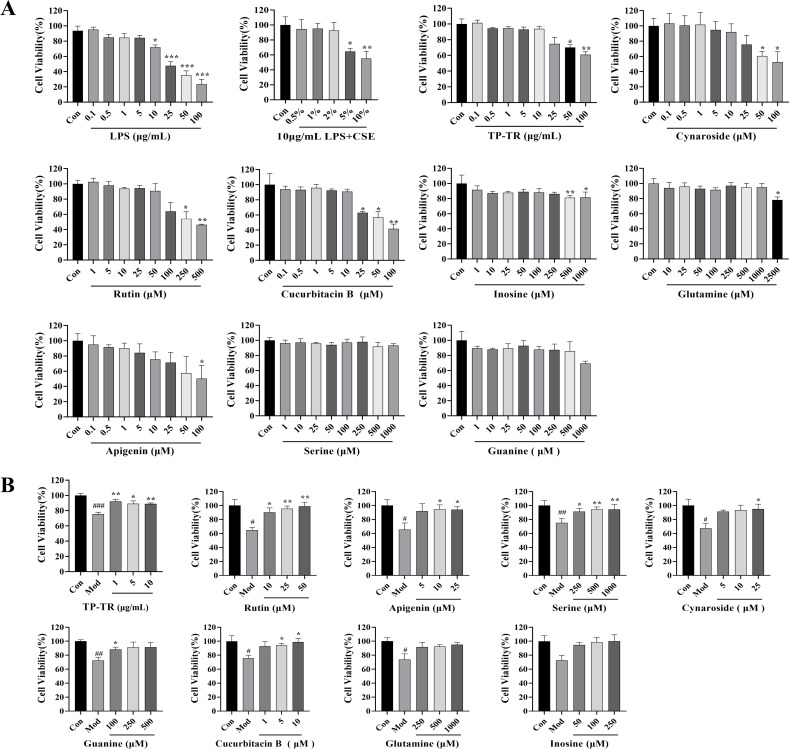
Effect of TP-TR and its chemical components on cell viability. **(A)** The cell viability of HBE135-E6E7 treated with LPS, CSE, TP-TR and eight chemical components. **(B)** Effect of TP-TR and eight chemical components on cell viability of model cells. Data are presented as means ± SEM, n = 3. ^#^*P* < 0.05, ^##^*P* < 0.01, ^###^*P* < 0.001, Mod VS Con; *P < 0.05, **P < 0.01, ***P < 0.001, Administration VS Mod.

The correlation analysis results of the TP-TR chemical components, gut microbial metabolites, and metabolites of lung and serum are shown in S9–S10 Figs. Rutin, cynaroside, cucurbitacin B, inosine, L-serine, glutamine, guanine, and apigenin were also screened. We assessed the cytotoxicity of various substances in vitro using the human bronchial epithelial cell line HBE135-E6E7. Cells were treated for 24 h with different concentrations of LPS, CSE, TP-TR, and the eight active ingredients. The results (Fig 8A) indicated that treatment with 10 μg/mL LPS combined with 5% CSE reduced cell viability to 35.5% of the control group, a statistically significant difference (*P* = 0.0133). In the TP-TR group, cell viability significantly decreased when drug concentrations exceeded 50 µg/mL. The cucurbitacin B group led to a notable reduction at concentrations greater than 25 µM. In the cynaroside group, cell viability showed a marked decrease when concentrations exceeded 50 µM, and the apigenin group showed a notable decline at concentrations exceeding 100 µM. The rutin group exhibited significant reductions above 250 µM, and the inosine group showed a notable decline at concentrations exceeding 500 µM. Lastly, the guanine group showed a decreasing trend in cell viability when concentrations exceeded 1000 µM, while the glutamine group exhibited a significantly decrease at concentrations above 2500 µM. To further investigate the protective effects of the drug on the model cells, we selected low, medium, and high doses within the safe concentration range for co-treatment with LPS and CSE for 24 h. The results indicated that each drug group showed a significant improvement or a tendency toward recovery in cell viability compared to the model group, mitigating the reduction in viability caused by LPS and CSE (Fig 8B).

**Fig 9:**
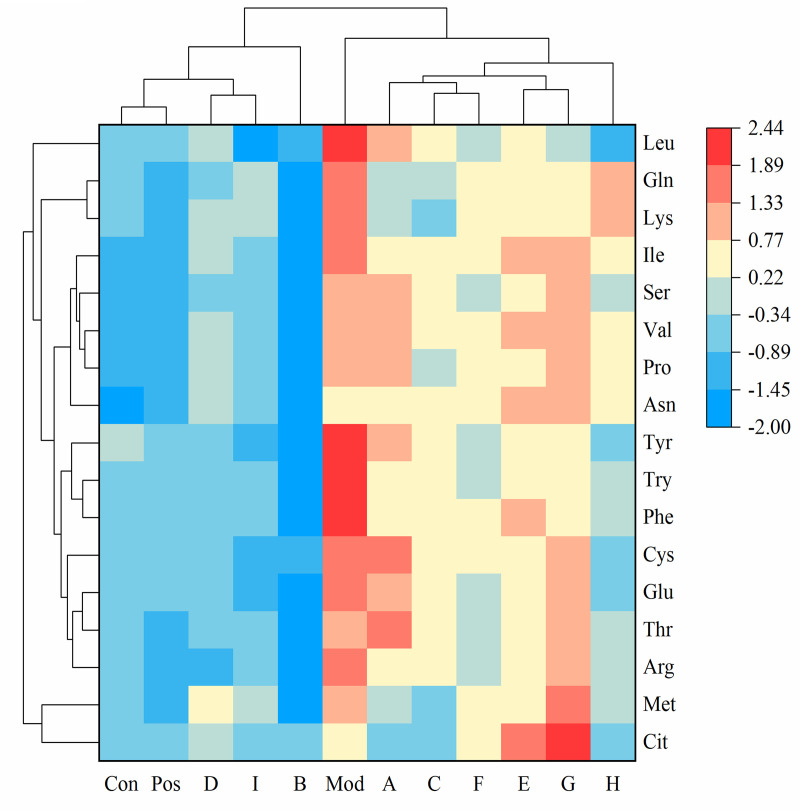
Analysis of the differential amino acids. A, Rutin; B, TP-TR; C, Cynaroside; D, Cucurbitacin B; E, Inosine; F, Serine; G, Glutamine; H, Guanine; I, Apigenin. (The red color represents up-regulated amino acids, while the blue color represents down-regulated amino acids.).

Furthermore, we analyzed the levels of 17 amino acids in these cells. To illustrate the differences in amino acid content across various samples, we standardized the amino acid data of each group and conducted cluster analysis. Specifically, each variable was scaled to a standard normal distribution via Z-score standardization (formula: =( − )/, where is the mean and is the standard deviation). The results are presented as a heat map (Fig 9). Compared with the control group, the model group showed increased levels of arginine, methionine, phenylalanine, tryptophan, tyrosine, valine, cysteine, isoleucine, lysine, and glutamine. Notably, the administration of TP-TR significantly restored the levels of the aforementioned 10 amino acids. Cucurbitacin B effectively reversed the levels of six amino acids: arginine, phenylalanine, tryptophan, tyrosine, cysteine, and glutamine. Luteoloside restores methionine, tyrosine, lysine, and glutamine levels. Apigenin reversed the effects of arginine, phenylalanine, tryptophan, tyrosine, and cysteine, whereas guanine reversed those of phenylalanine, tryptophan, tyrosine, and cysteine. Additionally, serine and glutamine effectively reversed tryptophan and tyrosine levels, whereas rutin and inosine restored tryptophan and tyrosine levels, respectively.

## Discussion

COPD is a complex disease with multiple phenotypes that affect the lung tissue and cardiovascular, gastrointestinal, and immune systems. Owing to its high morbidity and mortality, it has become a major disease worldwide [27]. The primary treatments for COPD are bronchodilators and anti-inflammatory drugs, which typically target a single mechanism of action. TCM emphasizes holistic regulation and comprehensively addresses COPD by improving lung function, reducing inflammation, and combating oxidative stress. Herb pairs, the smallest compatibility units in TCM prescriptions, simplify prescription analysis and improve clinical effectiveness and safety. The TP-TR herb pair originates from the renowned prescription “Beimu Gualou San” (Yi-Xue-Xin-Wu, Qing Dynasty), traditionally used for clearing heat, resolving phlegm, clearing the lungs, and relieving asthma. Master Shi Jinmo often combines Trichosanthis Pericarpium with Trichosanthis Radix to treat coughs caused by lung heat in clinical practice. Compared with complex prescriptions, herbal pairs maintain the benefits of compatibility while being more straightforward.

### TP-TR improves lung function, inhibits inflammation and oxidative stress, and delays lung tissue damage in COPD rats

Studies have shown that severe impairment of lung function coupled with a sharp increase in airway inflammation is an important indicator of the severity of COPD [28]. In clinical practice, increased respiratory frequency can preliminarily suggest an impact of the disease on respiratory function. In addition, airway resistance is significantly increased, and expiratory time is often prolonged due to airflow limitation in patients with COPD. In our study, changes in parameters such as Penh, PEF, MV, and TV provided important insights into pulmonary function status. Penh reflects airway resistance and was significantly elevated in model rats. The decrease in PEF indicated reduced airway patency. In addition, reductions in MV and TV suggested impaired gas exchange capacity, consistent with respiratory dysfunction in COPD. TP-TR improved pulmonary function in COPD rats by reducing bronchial resistance, increasing maximum expiratory flow and expiratory duration. Furthermore, inflammation and oxidative stress are two major pathogenic mechanisms in COPD patients. Many inflammatory cells are recruited from the bloodstream to the lungs under the guidance of locally released chemokines. TP-TR effectively reduced levels of inflammatory cytokines (IL-6, IL-17, IL-1β and TNF-α) and the oxidative stress marker MDA, while increasing SOD levels, thereby alleviating both inflammatory and oxidative damage. The chronic airway inflammation induced by COPD has been reported to be associated with the TLR4/NF-κB signaling pathway [29] and the TGF-β signaling pathway [30]. Additionally, the activation of macrophages can secrete various elastolytic enzymes MMP9 and MMP12, which can participate in the inflammatory response in COPD by degrading proteins and damaging the alveolar walls [30]. In our study, TP-TR alleviated airway inflammation and pulmonary fibrosis in COPD rats by reducing the expression of TLR4 and TGF-β. It also reduced the expression of MMP9/12 and MUC5AC/5B, thereby delaying lung tissue damage and reducing mucus production.

### TP-TR modulates gut microbiota and amino acid metabolism in COPD rats

Research has demonstrated the crosstalk between the gut and lungs, with the gut microbiota playing a key role in this process [19]. For example, *Lactobacillus rhamnosus* alleviates lung inflammation and structural damage in COPD mouse models, thereby improving lung function [31]. Additionally, clinical studies on patients with AECOPD have shown that the abundance of *Firmicutes* is low, whereas that of *Bacteroidetes* is high before treatment, indicating that gut dysbiosis may impair lung function by altering microecology and producing endotoxins [32]. There is also a complex interaction between TCM and the gut microbiota. On the one hand, TCM can regulate the composition and metabolism of gut microbiota. In contrast, intestinal microorganisms can metabolize and transform TCM compounds [33]. In this study, the TP-TR herbal pair increased the abundance of *Lactobacillus* and decreased the abundance of *g_ Anaerofustis*, *g_ Novosphingobium* and other genera. *Lactobacillus* exerts its effects by activating intestinal immune cells that migrate to the lungs and producing metabolites, such as SCFAs [34]. *Anaerofustis* may activate the secretion of proinflammatory cytokines through their metabolites or direct interactions with host immune cells [35]. *Novosphingobium* species are present in severe COPD and increase inflammation in mouse models of smoke exposure. Changes in these genera in the TP-TR group may help alleviate potential pro-inflammatory responses, thereby reducing inflammation levels.

TP-TR restored intestinal microecological balance by regulating microbial abundance. This change not only improved intestinal health but also produced metabolites that can affect the physiological state of the host through specific metabolic pathways. We analyzed the function of the gut microbiota in rats with COPD and primarily highlighted amino acid metabolic pathways. Additionally, an untargeted metabolomic analysis of rat serum and lung tissues demonstrated that exposure to cigarette smoke induced significant metabolic disturbances. The gut microbiota participates in host amino acid metabolism through multiple mechanisms and plays a key role in disease progression. Certain gut bacteria, such as *Morganella morganii* and some *Lactobacillus* species, can convert histidine into histamine via histidine decarboxylase. Microbial-derived histamine may influence pulmonary immunity by interacting with H1-H4 receptors on immune and structural cells in the lung, thereby promoting inflammation [36]. Moreover, gut microbes metabolize aromatic amino acids like phenylalanine and tyrosine into compounds including phenylacetic acid, phenyllactic acid, and p-cresol sulfate (PCS) [37]. Recent studies have shown that PCS promotes reactive oxygen species generation and oxidative stress across multiple organs, which may contribute to lung injury and inflammation [38].

Crucially, the functional capacity of the gut microbiota, dictated by the presence of specific bacterial enzymes such as decarboxylases, deaminases, and lyases, determines the metabolic fate of amino acids and the subsequent host response [18,39]. Untargeted metabolomics and metagenomics analyses in patients with COPD have revealed a significant negative correlation between the abundance of certain gut microbiota (such as *Streptococcus*) and fecal levels of amino acid metabolites including N-acetylglutamic acid and N-carbamoylglutamic acid. The gut microbiota may contribute to amino acid metabolic imbalance and thereby influence the progression of COPD through functional gene regulation, competition for metabolic substrates, or alteration of the intestinal microenvironment [40]. In the metabolism of aromatic amino acids, *Lactobacillus* converts tryptophan into indole-3-acetaldehyde (IAld), which activates the host aryl hydrocarbon receptor (AhR)-mediated IL-22 signaling pathway, thereby enhancing intestinal barrier integrity and suppressing systemic inflammation [41]. Further studies have shown that *Bacteroides* and *Clostridium* species can directly regulate host amino acid homeostasis by specifically metabolizing glutamate, tryptophan, and other amino acids [18]. These findings underscore the pivotal role of “gut microbiota-amino acid metabolism” in host physiological and pathological processes, involving mechanisms such as functional gene modulation, metabolite-mediated signaling pathways, and direct microbial metabolism. In our study, quantification of amino acid metabolites indicated that the TP-TR treatment restored tryptophan, valine, glutamic acid, methionine, phenylalanine, lysine, isoleucine, proline levels. Furthermore, *g_Anaerofustis, g_Novosphingobium, g_Christensenellaceae_R-7_group* and other genera were significantly correlated with amino acids. These findings further underscore the important role of amino acid metabolism in the mechanism by which TP-TR exerts its therapeutic effects in COPD.

Various therapeutic strategies targeting the gut-lung axis have shown promise in COPD management. Traditional Chinese medicine formulations, such as Bufei Jianpi granules and Shenqi Wenfei Formula, alleviate COPD symptoms by enhancing intestinal barrier function, restoring microbial balance (e.g., increasing *Lactobacillus*/*Bifidobacterium*, modulating *Parabacteroides*), and reducing lung inflammation [42–43]. Pharmacological interventions, including probiotics and antibiotics, regulate gut microbiota to mitigate pulmonary inflammatory responses [44]. Non-pharmacological approaches like high-fiber diets and aerobic exercise promote gut-derived anti-inflammatory SCFAs, benefiting lung tissue [45]. These findings highlight the gut-lung axis as a viable therapeutic target. TP-TR, as a multi-component herbal formulation, exhibits therapeutic effects comparable to other interventions by alleviating lung inflammation and oxidative stress while restoring gut microbial homeostasis.

### TP-TR bioactive compounds modulate amino acid metabolism and correlate with inflammation and oxidative stress in COPD cell model

Cucurbitacin B is considered one of the key active compounds of TP, and it can relieve chronic airway inflammation in COPD by inhibiting the production of inflammatory cytokines [46]. Meanwhile, flavonoids such as apigenin and luteolin in TP-TR also play a crucial role in antioxidant activity [47]. Moreover, glutamine has been shown to influence collagen production, slowing pulmonary fibrosis development. It also alleviates LPS-induced oxidative stress in lung tissue and reduces inflammatory factors like TNF-α in acute lung injury rats [48]. In this study, nucleosides and amino acids such as inosine, glutamine, and serine have a high correlation with inflammatory factors like IL-17 and TNF-α, suggesting that the increase in these components may help alleviate the inflammatory state, inhibit the leakage of inflammatory factors in COPD rats, and possibly modulate immune responses to reduce airway inflammation. Flavonoid compounds such as apigenin and cynaroside were also correlated with changes in oxidative stress markers like SOD and MDA. This suggests that these flavonoids may enhance antioxidant enzyme activity and reduce lipid peroxide formation, effectively alleviating oxidative damage and improving the oxidative stress state in the COPD model.

Amino acids serve as foundational elements in numerous metabolic pathways, and imbalances in these pathways are closely linked to inflammation and airway remodeling in COPD [14]. Our results demonstrated significant upregulation of arginine, methionine, phenylalanine, tryptophan, tyrosine, valine, cysteine, isoleucine, lysine, and glutamine in the model cells. Alterations in these amino acids may indicate a compensatory cellular adaptation to inflammation and oxidative stress, and may also be involved in more intricate metabolic pathways.

In the pathogenesis of COPD, the arginine-nitric oxide (NO) pathway is associated with pulmonary vascular function and airway inflammation [49–50]. Arginine can be catalyzed by nitric oxide synthase (NOS) to produce NO, a signaling molecule that not only participates in airway smooth muscle relaxation but also inhibits inflammation by regulating cytokine release. During chronic inflammation, the expression of inducible NOS (iNOS) in pulmonary tissues is dysregulated, potentially leading to impaired NO synthesis. On one hand, insufficient NO production may compromise the relaxation of airway smooth muscle, leading to increased airway resistance [51]; on the other hand, excessive NO and its derivatives may induce oxidative stress and cellular damage [52]. Therefore, persistent inflammation in COPD may drive cells to enhance arginine uptake and synthesis to regulate NO levels, which is supported by our observation of a significant upregulation of arginine in the model, indicating the presence of this compensatory mechanism. Moreover, during inflammation, pro-inflammatory factors such as IFN-γ can induce the expression of IDO1, accelerating the metabolism of tryptophan through the kynurenine pathway [53]. This metabolic pathway not only depletes tryptophan and inhibits T-cell proliferation, but also regulates oxidative stress and the immune microenvironment through its metabolites. Consequently, activation of IDO1 is considered a negative feedback anti-inflammatory mechanism. However, prolonged high expression of IDO1 may lead to immune suppression and limited tissue repair, thereby promoting the chronic progression of COPD [53]. We observed elevated tryptophan levels in the model cells, which may reflect abnormal IDO1 pathway function or blockage of downstream metabolism, suggesting that this pathway may play a complex regulatory role in the pathogenesis of COPD.

In addition, the host counteracts oxidative stress resulting from elevated levels of reactive oxygen species (ROS) by synthesizing antioxidants such as glutathione. Cysteine, glutamic acid, and glycine are the important substrates for GSH synthesis [54]. In this study, the upregulation of cysteine and glutamine may alleviate this oxidative stress by promoting the synthesis of glutathione, especially through the Nrf2 signaling pathway [55]. Methionine is pivotal for protein synthesis and cellular metabolism. Under conditions of inflammation and oxidative stress induced by COPD, cells may initiate repair and adaptation mechanisms that elevate methionine uptake and utilization. Additionally, cells may activate stress response pathways in which phenylalanine is metabolized into bioactive molecules such as tyrosine. Tyrosine is involved in stress regulation and cellular signal transduction [56] and its phosphorylation may activate the JAK-STAT pathway to regulate the cellular stress response and physiological functions [57]. Therefore, the total amount of tyrosine in the model cells may be increased to meet these requirements. After TP-TR treatment, we observed the restoration of the disturbed state of amino acid metabolism. Among the eight identified chemical components, cucurbitacin B, cynaroside, apigenin, guanine, and serine demonstrated restorative effects on various amino acids, corroborating our experimental findings.

### Summary and innovation of the study

**Fig 10:**
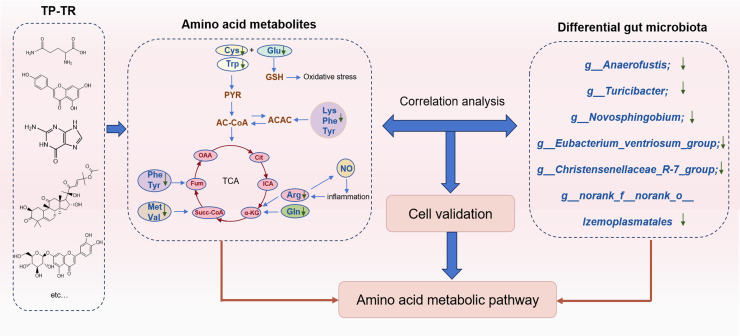
Schematic diagram illustrating the Trichosanthis Pericarpium – Trichosanthis Radix herbal pair’s role in alleviating COPD via gut microbiota and amino acid metabolism.

The study innovatively explores the compatibility and mechanism of action of TP – TR herbal pair in treating COPD, integrating pharmacodynamics, gut microbiota, and metabolomics analysis (Fig 10). TP-TR significantly improved pulmonary function in COPD rats, along with reduced levels of pro-inflammatory cytokines (such as IL-6, TNF-α), attenuated pulmonary infiltration, and decreased oxidative stress markers (e.g., MDA). Mechanistically, TP-TR downregulated the TLR4/TGF-β level to alleviate airway inflammation and pulmonary fibrosis, concurrently inhibiting MMP9/12 and MUC5AC/5B expression, thereby delaying lung tissue destruction and reducing mucus production. Importantly, this study innovatively demonstrates that TP-TR modulates the gut microbiota by enriching beneficial genera (e.g., *Lactobacillus*) and suppressing pro-inflammatory genera (e.g., *Anaerofustis, Novosphingobium*), suggesting its potential modulation of pulmonary inflammation through microbiota metabolism. Furthermore, TP-TR restored the levels of key amino acids such as tryptophan in COPD rats. Combined with cellular validation, we identified active chemical constituents such as cucurbitacin B, cynaroside, apigenin, guanine, and serine as potential regulators of amino acid metabolism disturbances in COPD cell model.

Our study innovatively provides the experimental evidence that the TP-TR herbal pair exerts therapeutic effects on COPD through bidirectional regulation of the gut-lung axis, establishing a novel mechanistic link between gut microbiota remodeling and pulmonary homeostasis restoration. Unlike previous studies that focused on lung-localized anti-inflammatory effects, this work highlights a multi-systemic, multi-targeted mechanism, offering a new perspective for understanding and utilizing classical herbal pairs in chronic respiratory diseases. These findings not only validate the clinical potential of TP-TR but also lay a solid foundation for its translational application, where safety evaluation and dose optimization in humans warrant further investigation.

### Study limitations

While this study provides important findings into the therapeutic effects of TP-TR on COPD, several limitations need to be acknowledged. Firstly, the smoke exposure combined with LPS tracheal instillation induced COPD in rats, which mimics some pathological features of human COPD, such as airway inflammation and emphysema. However, human COPD typically arises from long-term environmental exposures, such as smoking and air pollution, and involves a complex progression with systemic metabolic disturbances, comorbidities, and multi-organ interactions. Our model may not fully replicate the chronic low-grade inflammation and metabolic remodeling seen in patients, potentially leading to an underestimation of the sustained microbial dysbiosis and metabolic disruptions. Additionally, the metabolic disturbances in human COPD, particularly in amino acid metabolism, may involve more intricate compensatory mechanisms, which may not be fully captured by the animal model under the relatively short-term experimental conditions.

Moreover, species-specific differences in gut microbiome composition between rats and humans may limit the generalizability of our findings. The specific bacterial taxa regulated by TP-TR intervention in the animal model may not be directly translatable to human patients, where the microbiome composition could differ significantly. Furthermore, environmental and host factors that influence the human gut microbiome, such as diet, age, genetic background, and medication history, are absent in the highly controlled conditions of animal experiments. These factors could potentially mask the impact of individual variability in human clinical settings.

In addition, the safety profile of TP-TR components warrants attention, especially in the context of clinical application. Although TP-TR shows therapeutic potential, certain constituents such as cucurbitacin B (CuB) may raise safety concerns. CuB reduced cell viability at 25 μM, equivalent to 13.96 μg/g of crude drug. Given its low oral bioavailability in rats (1.37%) [58] and its content in TP-TR (450.97 μg/g), the estimated absorbed dose (~6.18 μg/g) is below the cytotoxic threshold. This suggests acceptable safety at standard doses. However, due to CuB’s narrow safety margin, clinical translation should proceed cautiously, with further in vivo toxicity studies and dose optimization to ensure safe long-term use in COPD patients.

Lastly, while our study highlighted the correlation between specific microbial taxa and metabolic changes, it did not establish a direct causal relationship between microbial communities and metabolic pathways. Future studies should further explore the interactions between microbial communities regulated by TP-TR and related metabolic pathways, aiming to identify key enzymes or metabolites involved. Fecal microbiota transplantation experiments, where microbiota from treated animals is transplanted into germ-free COPD models, could also provide valuable insights into the causality of microbiome function in disease improvement.

Collectively, these limitations suggest that while TP-TR shows promise in preclinical models, its clinical applicability remains to be established. Future studies should adopt more human-relevant models (e.g., chronic exposure models, aged animals, or organoids), integrate patient-derived microbiome data, and include stratification based on smoking history or disease stage. Such approaches will be essential for evaluating the real-world translational potential and individualized therapeutic effects of TP-TR in COPD management.

## Conclusion

TP-TR alleviates airway inflammation and pulmonary fibrosis in rats with COPD, reduces airway mucus secretion, and improves lung function. In addition, it helps alleviate oxidative stress. TP-TR may influence the composition of the gut microbiota and the levels of metabolites in COPD rats through amino acid metabolism. Through cell validation experiments, we found that TP-TR and its chemical constituents, cucurbitacin B, cynaroside, apigenin, guanine, and serine, regulated the metabolism of several amino acids.

## Supporting information

S1 File Contains Supporting Tables S1-S10 and Text S1. (DOCX)

S1 Fig Immunofluorescence analysis of MMP9, TLR4, and TGF-β. (PDF)

S2 Fig Gut microbiota of richness and diversity in rat. (PDF)

S3 Fig Differential analysis of gut microbiota among different groups. (PDF)

S4 Fig COG function classification of gut microbiota in rats. (PDF)

S5 Fig OPLS-DA scoring map. (PDF)

S6 Fig Relative peak areas of potential markers in serum and lung tissue in positive and negative ion mode. (PDF)

S7 Fig Determination results of amino acid components in rat serum and lung tissue. (PDF)

S8 Fig Heat map of correlation coefficient. (PDF)

S9 Fig Heat map of correlation coefficient between Intestinal flora index and component measurement index of Trichosanthis Pericarpium – Trichosanthis Radix herbal pair used to intervene COPD rats. (PDF)

S10 Fig Heat map of correlation coefficient between effect index, differential metabolites and component measurement index of Trichosanthis Pericarpium – Trichosanthis Radix herbal pair used to intervene COPD rats. (PDF)

S11 Fig The chemical profiles of flavonoids and amino acids in TP-TR. (PDF)

## Abbreviations

COPD: chronic obstructive pulmonary disease
CSE: cigarette smoke extract
HE: hematoxylin-eosin
IL-1β: Interleukin-1β
IL-6: Interleukin-6
KGS: keratinocyte growth supplement
LPS: lipopolysaccharide
MUC5AC: Mucin 5 AC
MUC5B: Mucin 5B
MMP9: Matrix metalloproteinase 9
MMP12: Matrix metalloproteinase 12
MTT: 3-[4,5-Dimethylthiazol-2-yl] 2,5-diphenyltetrazolium bromide
MDA: malondialdehyde
SOD: superoxide dismutase
TP: Trichosanthis Pericarpium
TR: Trichosanthis Radix
TNF-α: tumor necrosis factor-α
TLR4: Toll-Like receptor 4
TGF-β: transforming growth factor-β
TCM: traditional Chinese medicine
