# Supplementary material for: Investigating the Trichosanthis Pericarpium - Trichosanthis Radix herbal pair’s role in alleviating COPD through gut microbiota function, metabolomics analysis and cell validation experiment
Source: PLoS One. 2025 Aug 22;20(8):e0330621. doi: 10.1371/journal.pone.0330621 (PMC12373185; doi:10.1371/journal.pone.0330621)
Supplement: S1 File — (DOCX) [file pone.0330621.s001.docx]

Supplementary Materials

**Supplementary Table captions**

**S1 Table** Real-time PCR Primers.

**S2 Table** Retention time and related MS data of the amino acid components detected on the UHPLC-MS/MS.

**S3 Table** The analyte content from Trichosanthis Pericarpium - Trichosanthis Radix (TP - TR) and linear regression data and validation for reference compounds, precision, repeatability, stability, and recovery of the investigated compounds.

**S4 Table** Effect size and confidence interval of key indicators.

**S5 Table** Key Prediction of KEGG metabolic function of community samples.

**S6 Table** The 33 potential biomarkers identified in serum and lung tissue.

**S7 Table** Construction of metabolic pathways for potential differential metabolites in COPD rats.

**S8 Table** Retention time and related MS data of the flavonoid components in TP-TR detected on the UHPLC-MS/MS.

**S9 Table** Retention time and related MS data of the amino acid components in TP-TR detected on the UHPLC-MS/MS.

**S10 Table** Instrument model/manufacturer information table.

**S1 Table**. Real-time PCR Primers.

| Gene | Primers | number of bases |
| --- | --- | --- |
| GAPDH | F: AGCAATGCCTCCTGCACCACCAA | 26 |
|  | R: GCGGCCATCACGCCACAGTTT | 24 |
| MUC5AC | F: TCAACGGAGACTGCGAGTACAC | 22 |
|  | R: TCTTGATGGCCTTGGAGCA | 19 |
| MUC5B | F: CTGCGAGACCGAGGTCAACATC | 22 |
|  | R: TGGGCAGCAGGAGCACGGAG | 20 |
| TLR4 | F: CTCTGCCCTGCCACCATTTA | 20 |
|  | R: CTGGTCTCAGGCAGGAAAGG | 20 |
| TGF-β1 | F: TACAGGGCTTTCGCTTCAGT | 20 |
|  | R: TGGTTGTAGAGGGCAAGGAC | 20 |
| MMP-9 | F: TTGACAGCGACAAGAAGTGG | 20 |
|  | R: CCCTCAGTGAAGCGGTACAT | 20 |
| MMP-12 | F: ATGAAGCGTGCGGATGTAGACT | 22 |
|  | R: GAAATGTGTTGGGGTGAAGGTATC | 24 |

**S2 Table**. Retention time and related MS data of the nucleoside and amino acid components detected on the UHPLC-MS/MS.

| **Number** | **Name** | **tR/min** | **Q1 Mass(Da)** | **Q3 Mass(Da)** | **DP** | **EP** | **CE** | **CXP** | **Ion mode** |
| --- | --- | --- | --- | --- | --- | --- | --- | --- | --- |
| 1 | 2-L-Phenylalanine | 3.31 | 199.944 | 153.9 | 146 | 10 | 17 | 10 | ESI+ |
| 2 | Phenylalanine | 3.79 | 166.097 | 120.1 | 1 | 10 | 17 | 14 | ESI+ |
| 3 | Isoleucine | 3.79 | 132.07 | 86.2 | 11 | 10 | 13 | 10 | ESI+ |
| 4 | Tryptophan | 3.89 | 205.089 | 146.1 | 26 | 10 | 25 | 16 | ESI+ |
| 5 | Leucine | 4.13 | 132.069 | 69 | 11 | 10 | 25 | 8 | ESI+ |
| 6 | Methionine | 4.65 | 150.117 | 104 | 36 | 10 | 15 | 12 | ESI+ |
| 7 | Valine | 5.14 | 118.125 | 72 | 1 | 10 | 13 | 8 | ESI+ |
| 8 | Proline | 5.33 | 116.167 | 70 | 21 | 10 | 19 | 10 | ESI+ |
| 9 | Tyrosine | 5.61 | 182.097 | 136.1 | 41 | 10 | 19 | 16 | ESI+ |
| 10 | Cysteine | 6.08 | 122.001 | 76 | 56 | 10 | 17 | 10 | ESI+ |
| 11 | Threonine | 8.17 | 120.128 | 74 | 16 | 10 | 13 | 8 | ESI+ |
| 12 | Glutamic Acid | 9.14 | 148.051 | 84.1 | 66 | 10 | 21 | 10 | ESI+ |
| 13 | Lysine | 9.68 | 147.11 | 84 | 61 | 10 | 23 | 10 | ESI+ |
| 14 | Glutamine | 9.72 | 147.072 | 84.1 | 51 | 10 | 23 | 10 | ESI+ |
| 15 | Serine | 9.75 | 105.991 | 60 | 51 | 10 | 15 | 8 | ESI+ |
| 16 | Asparagine | 10.13 | 133.111 | 74 | 1 | 10 | 19 | 32 | ESI+ |
| 17 | Citrulline | 11.23 | 176.104 | 113 | 26 | 10 | 21 | 14 | ESI+ |
| 18 | Arginine | 16.45 | 175.11 | 70.1 | 86 | 10 | 27 | 8 | ESI+ |

**S3 Table** The analyte content from Trichosanthis Pericarpium - Trichosanthis Radix (TP - TR) and linear regression data and validation for reference compounds, precision, repeatability, stability, and recovery of the investigated compounds.

| Analyte | Content (μg/g, n=3) | Regression equation | R^2^ | Linear range (μg/mL) | LOD (ng) | LOQ (ng) | Precision (RSD, %) | | | | | | Repeatability (RSD, %, n = 6) | Stability (RSD, %, n = 6) | Recovery  (%, n =3) | |
| --- | --- | --- | --- | --- | --- | --- | --- | --- | --- | --- | --- | --- | --- | --- | --- | --- |
|  |  |  |  |  |  |  | Intra-day (*n* = 6) | | | Inter-day (*n* = 6) | | |  |  |  |  |
|  |  |  |  |  |  |  | Low | Medium | High | Low | Medium | High |  |  | mean | RSD (%) |
| quercetin | 0.05±0.03 | y=23137x+267.75 | 0.9954 | 0.0474-7.59 | 0.01 | 0.02 | 0.33 | 1.54 | 0.78 | 3.01 | 2.36 | 3.25 | 3.53 | 3.91 | 96.22 | 0.93 |
| rutin | 26.26±5.34 | y=10002x+406.16 | 0.9996 | 0.33-16.5 | 0.06 | 0.2 | 0.84 | 1.93 | 0.94 | 2.14 | 2.09 | 3.47 | 3.65 | 3.98 | 98.11 | 0.67 |
| luteolin | 1.49±0.41 | y=50848x+1562.7 | 0.9972 | 0.552-5.52 | 0.01 | 0.02 | 0.78 | 1.07 | 2.12 | 3.44 | 2 | 2.98 | 2.47 | 1.21 | 97.03 | 0.98 |
| isoquercitrin | 4.01±0.91 | y=9087x+707.91 | 0.9965 | 0.183-1.83 | 0.01 | 0.03 | 1.24 | 1.49 | 2.11 | 2.89 | 2.59 | 3.14 | 3.26 | 4.77 | 98.51 | 0.73 |
| cynaroside | 5.12±0.39 | y=12352x+1479 | 0.9971 | 0.896-1.12 | 0.16 | 0.53 | 1.33 | 2.05 | 1.09 | 2.45 | 2.15 | 2.04 | 2.37 | 1.53 | 98.2 | 0.89 |
| apigenin | 0.44±0.06 | y=48001x+1923.8 | 0.9993 | 0.343-3.43 | 0.03 | 0.11 | 1.47 | 1.35 | 2.81 | 3.07 | 1.6 | 2.22 | 2.06 | 0.19 | 96.74 | 1.23 |
| cucurbitacin D | 0.28±0.20 | y=52.124x-1.2725 | 0.9995 | 0.0591-5.91 | 0.05 | 0.18 | 2.04 | 3.59 | 2.45 | 2.58 | 3.86 | 4.02 | 3.51 | 3.91 | 98.16 | 3.11 |
| cucurbitacin B | 450.97±100.68 | y=27.478x+68.034 | 0.9932 | 18.3-183 | 0.06 | 0.19 | 2.01 | 3.61 | 1.22 | 3.07 | 3.58 | 2.14 | 2.55 | 3.31 | 96.7 | 0.87 |
| cucurbitacin E | 4.55±0.99 | y=80.558x-3.6915 | 0.9974 | 1.09-109 | 0.52 | 1.73 | 2.87 | 3.55 | 2.54 | 4.57 | 4.77 | 3.45 | 2.62 | 3.17 | 99.16 | 5.85 |
| L-threonine | 123.77±9.94 | y=14520x-7125.7 | 0.9976 | 0.672-67.2 | 0.22 | 0.75 | 1.57 | 3.04 | 2.44 | 3.59 | 4.2 | 2.58 | 2.16 | 4.6 | 100.98 | 1.52 |
| L-glutamine | 111.33±9.99 | y=55575x-39941 | 0.9965 | 0.842-84.2 | 0.04 | 0.13 | 2.01 | 2.25 | 1.25 | 4.04 | 3.79 | 2.74 | 2.65 | 2.43 | 97.57 | 2.26 |
| L-serine | 31.19±3.60 | y=7233.4x+5416.7 | 0.9977 | 0.725-7.25 | 0.12 | 0.42 | 1.55 | 4.47 | 2.87 | 4.24 | 3.74 | 2.79 | 4.21 | 4.86 | 99.34 | 3.29 |
| L-tyrosine | 105.67±12.26 | y=19645x+9359.6 | 0.9993 | 0.761-76.1 | 0.12 | 0.42 | 2.58 | 4.33 | 3.45 | 3.25 | 4.79 | 2.41 | 3 | 2.9 | 98.12 | 3.54 |
| L-lysine | 59.46±4.18 | y=74406x-35432 | 0.9977 | 0.726-72.6 | 0.1 | 0.32 | 2.48 | 3.44 | 3.21 | 2.14 | 3.57 | 2.11 | 3.88 | 2.24 | 102.56 | 1.02 |
| L-leucine | 12.88±0.66 | y=18380x+1209.4 | 0.9968 | 0.721-72.1 | 0.19 | 0.62 | 2.48 | 4.22 | 2.14 | 3.78 | 4.93 | 3.04 | 2.65 | 3.3 | 95.97 | 2.14 |
| γ-aminobutyric acid | 169.44±5.36 | y=14996x+3856.1 | 0.9958 | 0.803-80.3 | 0.05 | 0.17 | 1.84 | 2.17 | 1.44 | 2.57 | 3.42 | 2.98 | 4 | 2.26 | 102.34 | 1.55 |
| L-citrulline | 7901.47±321.73 | y=17903x+293.63 | 1 | 85.1-851.0 | 0.1 | 0.34 | 1.44 | 3.36 | 2.45 | 3.69 | 3.72 | 2.59 | 3.49 | 4.65 | 99.1 | 2.44 |
| L-valine | 110.5±10.06 | y=114678x+65062 | 0.9964 | 0.672-67.2 | 0.11 | 0.37 | 1.74 | 1.48 | 1.27 | 3.47 | 3.02 | 2.57 | 3.43 | 3.2 | 98.11 | 4.33 |
| L-tryptophan | 25.6±2.72 | y=17612x+3431.4 | 0.9969 | 0.723-72.3 | 0.04 | 0.14 | 2.51 | 2.5 | 1.45 | 3.75 | 4.88 | 2.39 | 3.1 | 1.91 | 95.79 | 1.47 |
| L-isoleucine | 40.3±3.37 | y=3547.9x+1408.8 | 0.9964 | 0.72372.3 | 0.1 | 0.32 | 2.87 | 4.09 | 3.24 | 3.47 | 4.88 | 3.18 | 1.7 | 1.46 | 103.74 | 2.85 |
| L-phenylalanine | 15.71±0.71 | y=209500x+28962 | 0.9976 | 0.842-84.2 | 0.02 | 0.07 | 0.89 | 1.79 | 2.45 | 2.87 | 1.99 | 3.01 | 1.45 | 1.36 | 101.25 | 7.47 |
| L-proline | 121.86±9.45 | y=27430x+1679.5 | 0.9971 | 0.672-67.2 | 0.13 | 0.43 | 2.58 | 3.41 | 1.41 | 2.57 | 4.69 | 3.47 | 2.97 | 2.06 | 103.67 | 0.88 |
| trans-4-hydroxy-L-proline | 7.32±0.47 | y=14644x-10393 | 0.9951 | 0.672-67.2 | 0.01 | 0.04 | 1.78 | 3.36 | 2.47 | 4.78 | 4.45 | 4.05 | 4.32 | 3.35 | 98.86 | 4.7 |
| L-methionine | 11.97±0.87 | y=54958x+12706 | 0.9984 | 0.724-72.4 | 0.04 | 0.13 | 1.47 | 1.71 | 1.24 | 2.58 | 4.58 | 3.21 | 2.98 | 4.05 | 100.59 | 0.32 |

**S4 Table.** Effect size and confidence interval of key indicators.

| Index | Comparison | Control vs Model | TP-TR-H vs Model |
| --- | --- | --- | --- |
| Pulmonary Function | Penh | Cohen's d=4.963(95%CI:[2.678,7.248]) | Cohen's d=5.469(95%CI:[3.006,7.932]) |
|  | PEF | Cohen's d=-3.902(95%CI:[-5.831,-1.974]) | Cohen's d=-5.589(95%CI:[-8.096,-3.083]) |
|  | TV | Cohen's d=-2.849(95%CI:[-4.455,-1.243]) | Cohen's d=-2.286(95%CI:[-3.741,-0.831]) |
|  | MV | Cohen's d=-6.066(95%CI:[-8.743,-3.388]) | Cohen's d=-6.13(95%CI:[-8.832,-3.429]) |
|  | F | Cohen's d=14.207(95%CI:[8.411,20.002]) | Cohen's d=8.846(95%CI:[5.13,12.561]) |
|  | Te | Cohen's d=-8.063(95%CI:[-11.481,-4.644]) | Cohen's d=-2.416(95%CI:[-3.904,-0.927]) |
| Inflammation-related factors | MMP9 | Cohen's d=9.391(95%CI:[5.467,13.314]) | Cohen's d=4.76(95%CI:[2.545,6.976]) |
|  | MMP12 | Cohen's d=7.489(95%CI:[4.286,10.692]) | Cohen's d=7.073(95%CI:[4.025,10.12]) |
|  | MUC5AC | Cohen's d=2.021(95%CI:[0.63,3.412]) | Cohen's d=2.761(95%CI:[1.18,4.342]) |
|  | MUC5AB | Cohen's d=9.162(95%CI:[5.326,12.998]) | Cohen's d=7.191(95%CI:[4.1,10.283]) |
|  | TGF-β | Cohen's d=8.417(95%CI:[4.864,11.969]) | Cohen's d=9.471(95%CI:[5.516,13.426]) |
|  | TLR4 | Cohen's d=6.329(95%CI:[3.556,9.103]) | Cohen's d=6.904(95%CI:[3.919,9.889]) |
|  | IL-6 | Cohen's d=4.8(95%CI:[2.571,7.029]) | Cohen's d=3.889(95%CI:[1.965,5.813]) |
|  | IL-17 | Cohen's d=1.912(95%CI:[0.546,3.278]) | Cohen's d=2.292(95%CI:[0.836,3.749]) |
|  | IL-1β | Cohen's d=1.235(95%CI:[0,2.47]) | Cohen's d=2.154(95%CI:[0.731,3.576]) |
|  | TNF-α | Cohen's d=4.076(95%CI:[2.091,6.061]) | Cohen's d=3.172(95%CI:[1.472,4.873]) |
| Oxidative stress indicators | SOD | Cohen's d=-4.517(95%CI:[-6.65,-2.385]) | Cohen's d=-4.443(95%CI:[-6.55,-2.336]) |
|  | MDA | Cohen's d=4.96(95%CI:[2.676,7.245]) | Cohen's d=1.949(95%CI:[0.575,3.324]) |

**S5 Table**. Key Prediction of KEGG metabolic function of community samples.

| **First-level functional layer** | **Secondary functional layer** |
| --- | --- |
| Metabolism | Amino Acid Metabolism |
|  | Biosynthesis of Other Secondary Metabolites |
|  | Cancers |
|  | Carbohydrate Metabolism |
|  | Cardiovascular Diseases |
|  | Energy Metabolism |
| Cellular Processes | Cell Growth and Death |
|  | Cell Motility |
|  | Cellular Processes and Signaling |
| Organismal Systems | Circulatory System |
|  | Digestive System |
|  | Endocrine System |
|  | Environmental Adaptation |
|  | Enzyme Families |
|  | Excretory System |
| Genetic Information Processing | Folding, Sorting and Degradation |
|  | Genetic Information Processing |

**S6 Table**. The 33 potential biomarkers identified in serum and lung tissue.

| **No.** | **Metabolites** | **TR(min)** | **Mass(m/z)** | **Formula** | **Trend** | **HMDB ID** | **Ion mode** | **KEGG ID** | **Adduct** | **ppm** | **Source** |
| --- | --- | --- | --- | --- | --- | --- | --- | --- | --- | --- | --- |
| PM1 | (6Z,9Z)-tetradeca-6,9-dienoic acid | 8.6 | 83.0592 | C14H24O2 | ↓ | 0340799 | + | NA | M+2H+Na | 15 | serum |
| PM2 | LysoPC（16：0） | 8.78 | 496.3298 | C_24_H_50_NO_7_P | ↑ | 0010382 | + | NA | M+H | 2 | serum |
| PM3 | Glyceryl 5-hydroxydecanoate | 10.69 | 525.3633 | C_13_H_26_O_5_ | ↑ | 0032297 | + | NA | 2M+H | 0 | serum |
| PM4 | LysoPC（18：2（9Z，12Z）/ 0：0） | 8.06 | 520.3298 | C_26_H_50_NO_7_P | ↑ | 0010386 | + | C04230 | M + H | 1 | serum |
| PM5 | PC(PGE2/DiMe(13,5)) | 8.78 | 497.3325 | C_52_H_90_NO_12_P | ↑ | 0289138 | + | NA | M+ACN+2H | 4 | serum |
| PM6 | LysoPC(0:0/20:4(5Z,8Z,11Z,14Z)) | 8.42 | 544.3316 | C_28_H_50_NO_7_P | ↑ | 0061699 | + | NA | M + H | 0 | serum |
| PM7 | (10Z,12E)-tetradeca-10,12-dienoic acid | 6.90 | 83.0593 | C_14_H_24_O_2_ | ↓ | 0340798 | + | NA | M+2H+Na | 14 | serum |
| PM8 | LysoPE(20:1(11Z)/0:0) | 10.29 | 525.3641 | C_25_H_50_NO_7_P | ↑ | 0011512 | + | NA | M+NH_4_ | 4 | serum |
| PM9 | LysoPE(18:1(11Z)/0:0) | 8.43 | 497.3331 | C_23_H_46_NO_7_P | ↑ | 0011505 | + | NA | M+NH_4_ | 4 | serum |
| PM10 | Deterrol stearate | 9.21 | 523.3481 | C_33_H_50_O_2_ | ↑ | 0034578 | + | NA | M+2Na-H | 8 | serum |
| PM11 | Phenyl-Alanine | 2.10 | 188.0685 | C9H11NO2 | ↑ | 0256428 | + | NA | M+Na | 2 | serum |
| PM12 | 9Z-Eicosenoic acid | 5.73 | 355.2588 | C_20_H_38_O_2_ | ↑ | 0062436 | + | NA | M+2Na-H | 1 | serum |
| PM13 | LysoPC(18：2（9Z，12Z）/ 0：0) | 3.08 | 564.3510 | C_26_H_50_NO_7_P | ↑ | 0010386 | + | NA | M + FA-H | 1 | serum |
| PM14 | Dehydrocarpaine I | 8.06 | 521.3326 | C_28_H_48_N_2_O_4_ | ↑ | 0030271 | + | NA | M+2Na-H | 0 | serum |
| PM15 | (4-Aminophenyl)phosphonic acid | 2.50 | 98.5109 | C_6_H_8_NO_3_P | ↑ | 0243614 | + | NA | M+H+Na | 6 | serum |
| PM16 | 18-Hydroxyarachidonic acid | 9.78 | 303.2274 | C_20_H_32_O_3_ | ↑ | 0006245 | + | NA | M + H-H_2_O | 2 | serum |
| PM17 | LysopPC[18:1(9Z)] | 9.47 | 522.3481 | C_26_H_52_NO_7_P | ↑ | 0002815 | + | C04230 | M+H | 1 | serum |
| PM18 | Cholesterol sulfate | 8.13 | 545.3318 | C_27_H_46_O_4_S | ↑ | 0000653 | + | C18043 | M+DMSO+H | 2 | serum |
| PM19 | Neohesperidoside | 2.12 | 205.0946 | C_12_H_22_O_10_ | ↑ | 0302540 | + | C08244 | M+2ACN+2H | 1 | serum |
| PM20 | Hexa-D-arginine | 8.78 | 498.3348 | C_36_H_75_N_25_O_6_ | ↑ | 0253116 | + | NA | M+ACN+2H | 5 | serum |
| PM21 | SM(d17:1/24:1(15Z)) | 7.21 | 400.3372 | C_46_H_91_N_2_O_6_P | ↑ | 0011696 | + | NA | M+2H | 2 | serum |
| PM22 | LysoPC（17：0/0：0） | 9.56 | 508.3692 | C_25_H_52_NO_7_P | ↑ | 0012108 | + | C04230 | M-H | 1 | serum |
| PM23 | Palmitic acid | 4.46 | 274.2708 | C16H32O2 | ↑ | 0000220 | + | C00249 | M+NH4 | 12 | serum |
| PM24 | LysoPC(16:1(9Z)/0:0) | 9.68 | 511.3508 | C_24_H_48_NO_7_P | ↑ | 0010383 | + | C04230 | M+NH4 | 0 | serum |

**S6 Table continued**. The 33 potential biomarkers identified in serum and lung tissue.

| PM25 | LysoPC（18：1（11Z）/ 0：0） | 7.87 | 588.3136 | C_26_H_52_NO_7_P | ↑ | 0010385 | - | C04230 | M-H + HCOONa | 1 | serum |
| --- | --- | --- | --- | --- | --- | --- | --- | --- | --- | --- | --- |
| PM26 | Lithocholyltaurine | 7.87 | 528.2943 | C_26_H_45_NO_5_S | ↑ | 0000722 | - | C02592 | M + FA-H | 17 | serum |
| PM27 | (R+)-3-(4-hydroxyphenyl)lactate | 2.17 | 188.0679 | C_9_H_9_O_4_ | ↓ | 0300933 | + | NA | M+Li | 7 | lung tissue |
| PM28 | PE(20:5(5Z,8Z,11Z,14Z,17Z)/16:1(9Z)) | 2.02 | 246.1669 | C_41_H_70_NO_8_P | ↓ | 0009452 | + | NA | M+3H | 7 | lung tissue |
| PM29 | DL-Proline | 1.44 | 120.0791 | C_5_H_9_NO_2_ | ↓ | 0251528 | + | C16435 | M+3ACN+2H | 3 | lung tissue |
| PM30 | Norsalsolinol | 1.39 | 166.0840 | C_9_H_11_NO_2_ | ↓ | 0006044 | + | NA | M+H | 0 | lung tissue |
| PM31 | Methyl valine | 1.25 | 132.1005 | C_6_H_13_NO_2_ | ↓ | 0253926 | + | NA | M+H | 11 | lung tissue |
| PM32 | DL-Glutamate | 1.03 | 130.0482 | C_5_H_9_NO_4_ | ↑ | 0060475 | + | C00302 | M+H-H2O | 17 | lung tissue |
| PM33 | formate | 1.19 | 91.0011 | CHO_2_ | ↑ | 0304356 | - | NA | 2M+H | 16 | lung tissue |

| No. | Pathway Name | Match Status | p | -log(p) | Holm p | FDR | Impact | Details |
| --- | --- | --- | --- | --- | --- | --- | --- | --- |
| 1 | Glycerophospholipid metabolism | 2/36 | 0.01 | 2.13 | 0.62 | 0.62 | 0.12 | KEGG |
| 2 | Phenylalanine, tyrosine and tryptophan biosynthesis | 44565 | 0.02 | 1.81 | 1.00 | 0.65 | 0.50 | KEGG/SMP |
| 3 | Phenylalanine metabolism | 44571 | 0.04 | 1.42 | 1.00 | 1.00 | 0.36 | KEGG/SMP |
| 4 | Glycosylphosphatidylinositol (GPI)-anchor biosynthesis | 44575 | 0.05 | 1.28 | 1.00 | 1.00 | 0.00 | KEGG |
| 5 | Glyoxylate and dicarboxylate metabolism | 1/32 | 0.12 | 0.93 | 1.00 | 1.00 | 0.00 | KEGG |
| 6 | Biosynthesis of unsaturated fatty acids | 1/36 | 0.13 | 0.88 | 1.00 | 1.00 | 0.00 | KEGG |
| 7 | Fatty acid elongation | 1/39 | 0.14 | 0.85 | 1.00 | 1.00 | 0.00 | KEGG/SMP |
| 8 | Fatty acid degradation | 1/39 | 0.14 | 0.85 | 1.00 | 1.00 | 0.00 | KEGG/SMP |
| 9 | Fatty acid biosynthesis | 1/47 | 0.17 | 0.77 | 1.00 | 1.00 | 0.01 | KEGG/SMP |
| 10 | Aminoacyl-tRNA biosynthesis | 1/48 | 0.17 | 0.76 | 1.00 | 1.00 | 0.00 | KEGG |
| 11 | Steroid hormone biosynthesis | 1/85 | 0.29 | 0.54 | 1.00 | 1.00 | 0.00 | KEGG/SMP |

**S7 Table.** Construction of metabolic pathways for potential differential metabolites in COPD rats.

**S8 Table.** Retention time and related MS data of the flavonoid components in TP-TR detected on the UHPLC-MS/MS.

| Analytes | t_R_ (min) | Precursor ion (m/z) | Product ions (m/z) | Cone (V) | Collision (V) | Ion mode |
| --- | --- | --- | --- | --- | --- | --- |
| apigenin | 7.62 | 270.989 | 153.10,119.10 | 52 | 31 | ES- |
| cynaroside | 4.71 | 447.19 | **285.03** | 48 | 26 | ES- |
| cucurbitacin D | 7.69 | 515.41 | 165.05,479.33 | 32 | 36 | ES- |
| cucurbitacin B | 7.92 | 576.207 | 499.10,481.10 | 21 | 22 | ES- |
| cucurbitacin E | 7.99 | 555.41 | **495.36** | 26 | 14 | ES- |
| luteolin | 7.35 | 284.913 | 132.90,175.10,131.90 | 46 | 23 | ES- |
| quercetin | 7.19 | 301.08 | 150.94，179.01 | 28 | 17 | ES- |
| isoquercitrin | 4.93 | 463.16 | 300.18,271.09 | 38 | 28 | ES- |
| rutin | 4.91 | 608.99 | 299.80,300.30,270.90 | 52 | 31 | ES- |

| Analytes | t_R_ (min) | Precursor ion (m/z) | Product ions (m/z) | Cone (V) | Collision (V) | Ion mode |
| --- | --- | --- | --- | --- | --- | --- |
| L-threonine | 6.75 | 120.128 | 56.00, 74.00, 102.00 | 13 | 8 | ES+ |
| L-glutamine | 7.89 | 147.050 | 41.02 | 14 | 16 | ES+ |
| L-serine | 8.03 | 105.970 | 59.99, 60.00 | 14 | 8 | ES+ |
| L-tyrosine | 4.79 | 182.097 | 91.00, 123.00, 136.10 | 19 | 16 | ES+ |
| L-lysine | 7.89 | 147.090 | 84.05, 66.97 | 16 | 14 | ES+ |
| L-leucine | 2.83 | 132.070 | 43.83, 86.01 | 16 | 20 | ES+ |
| γ-aminobutyric acid | 3.17 | 104.000 | 42.97, 68.73 | 14 | 10 | ES+ |
| L-valine | 4.14 | 118.125 | 55.00, 72.00 | 13 | 8 | ES+ |
| L-tryptophan | 2.97 | 205.089 | 118.11, 143.00, 146.10 | 25 | 16 | ES+ |
| L-isoleucine | 3.11 | 132.010 | 41.02, 68.99 | 16 | 8 | ES+ |
| L-phenylalanine | 2.82 | 166.097 | 77.10, 103.00, 120.10 | 17 | 14 | ES+ |
| L-proline | 4.36 | 116.170 | 68.00, 68.10, 70 | 37 | 8 | ES+ |
| trans-4-hydroxy-L-proline | 6.27 | 132.000 | 67.95 | 18 | 12 | ES+ |
| L-methionine | 3.61 | 150.111 | 56.00, 61.00, 104.00 | 15 | 12 | ES+ |
| L-citrulline | 8.93 | 176.090 | 69.96, 113.08 | 14 | 14 | ES+ |

**S9 Table.** Retention time and related MS data of the amino acid components in TP-TR detected on the UHPLC-MS/MS.

**S10 Table** Instrument model/manufacturer information table

| **Instrument Name** | **Model Number** | **Manufacturer and Country** |
| --- | --- | --- |
| C100 fully automatic cigarette smoke generator | Y10032919 | Shanghai Yuyan Scientific Instrument Co., Ltd., China |
| Pulmonary solutions for small & large animals | EMKA-WBP | Emka Technologies, France |
| Real-time quantitative PCR system | ABI-7500 | Thermo Fisher Scientific, USA |
| Tissue homogenizer | Tissuelyser-48 | Shanghai Jingxin Industrial Development Co., Ltd., China |
| Microplate reader | Spectrum MAX190 | PerkinElmer, USA |
| CO₂ incubator | Thermo 3100 | Thermo Fisher Scientific, USA |
| Inverted microscope | TE-2000 | Nikon Corporation, Japan |
| Rotary evaporator | Buchi R-210 | Shanghai Jinfu Experimental Equipment Co., Ltd., China |
| Ultrasonic cleaner | KH-500 DV | Kunshan Hechuang Ultrasonic Instruments Co., Ltd., China |
| Centrifuge | Microfuge® 22R | Beckman Coulter, USA |
| Electronic analytical balances | ML204, MS105 | Mettler-Toledo Instruments Co., Ltd. |
| Ultra Performance Liquid Chromatography (UPLC) system | ACQUITY UPLC System | Waters Corporation, Milford, USA |
| Mass spectrometry detector | AB SCIEX Triple Quad 6500 plus | AB SCIEX, Massachusetts, USA |

**S1 Text Immunofluorescence staining method**

The paraffin-embedded lung tissue sections were dewaxed using deparaffinization solutions, followed by ethanol dehydration and washing with distilled water. Antigen retrieval was performed using citrate buffer under high-pressure heat (95°C, 15 minutes), with the slides washed three times in PBS (pH 7.4). For blocking, a circle was drawn around the tissue, and 10% BSA was applied, followed by a 30-minute incubation. The primary antibody was added and incubated overnight at 4°C. After washing (5 minutes, 3 times), the secondary antibody was applied and incubated for 50 minutes at room temperature in the dark. Nuclear staining was performed using DAPI for 10 minutes, followed by quenching of autofluorescence with a specific agent. Finally, the slides were mounted with an anti-fade medium. Fluorescence images were captured using specific excitation and emission wavelengths for DAPI and CY3. Fluorescence intensity was quantified with Image J.
