# Supplementary material for: Investigating the Trichosanthis Pericarpium - Trichosanthis Radix herbal pair’s role in alleviating COPD through gut microbiota function, metabolomics analysis and cell validation experiment
Source: PLoS One. 2025 Aug 22;20(8):e0330621. doi: 10.1371/journal.pone.0330621 (PMC12373185; doi:10.1371/journal.pone.0330621)
Supplement: S1 Fig — (PDF) [file pone.0330621.s002.pdf]

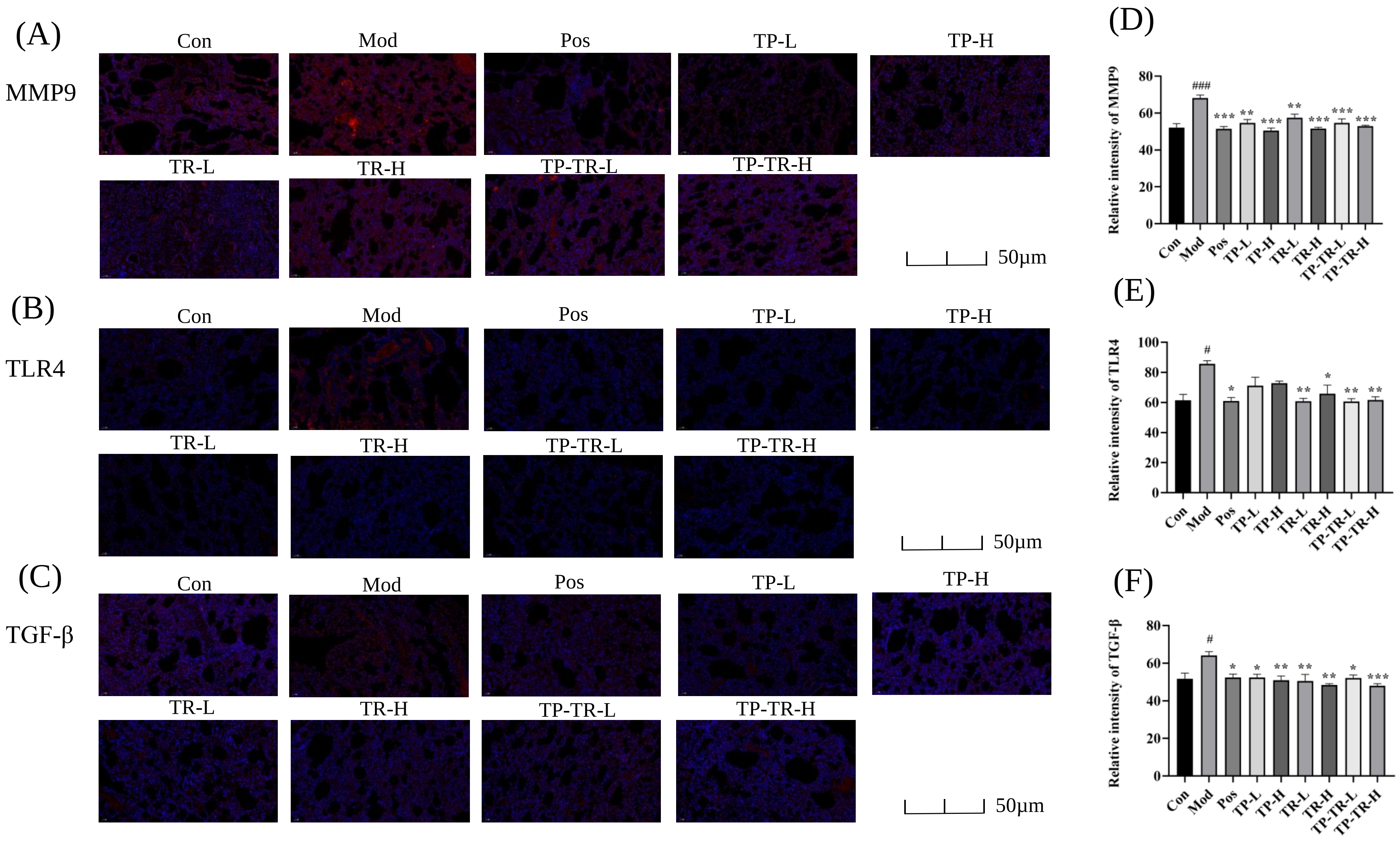


**S1 Fig.** Immunofluorescence analysis of MMP9, TLR4, and TGF-β. (A-C) Immunofluorescence staining of MMP9, TLR4, TGF-β; (D-F) The quantitative value of the relative fluorescence intensity of MMP9, TLR4, TGF-β.
