# Supplementary material for: Investigating the Trichosanthis Pericarpium - Trichosanthis Radix herbal pair’s role in alleviating COPD through gut microbiota function, metabolomics analysis and cell validation experiment
Source: PLoS One. 2025 Aug 22;20(8):e0330621. doi: 10.1371/journal.pone.0330621 (PMC12373185; doi:10.1371/journal.pone.0330621)
Supplement: S2 Fig — (PDF) [file pone.0330621.s003.pdf]

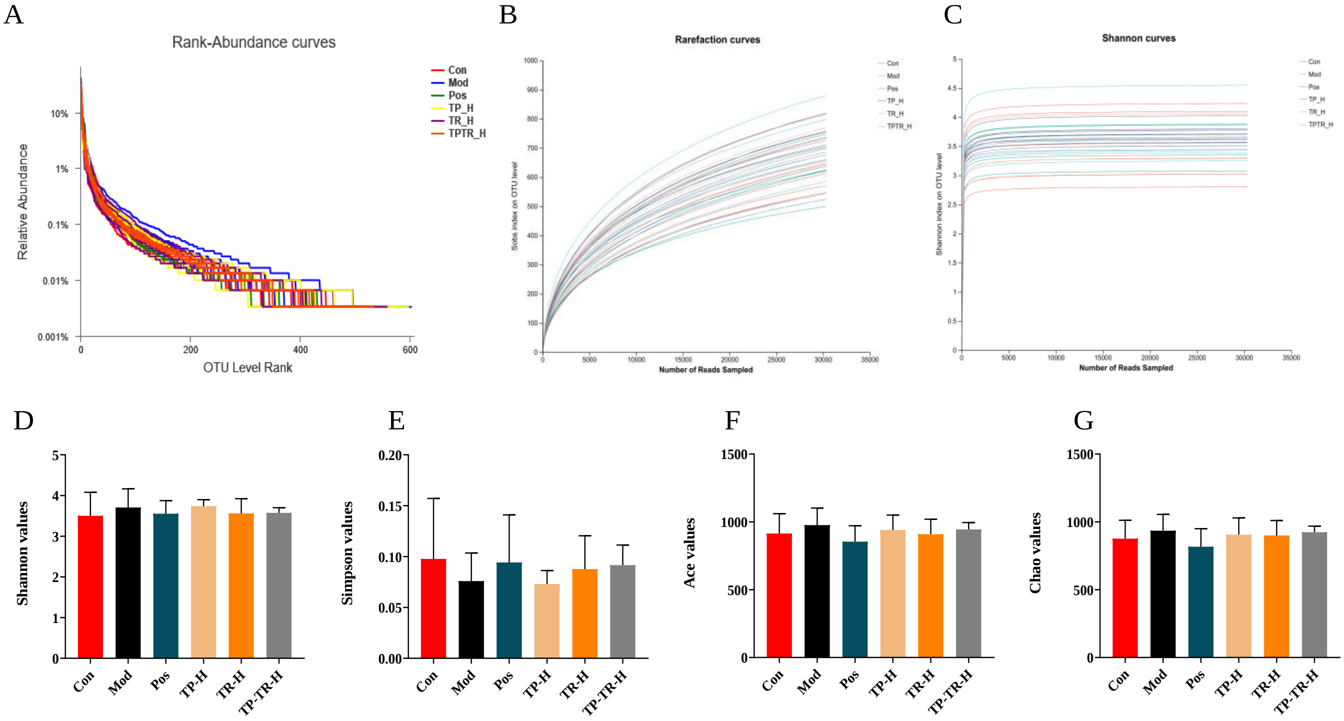

S2 Fig. Gut microbiota of richness and diversity in rat (A) Rank-abundance curve, (B) Rarefaction curve (Shannon-Wiener), (C) Rarefaction curve (Sobs) ; Gut microbiota of diversity in rat (D) Shannon, (E) Simpson, (F) Ace, (G) Chao.
