# Supplementary material for: Investigating the Trichosanthis Pericarpium - Trichosanthis Radix herbal pair’s role in alleviating COPD through gut microbiota function, metabolomics analysis and cell validation experiment
Source: PLoS One. 2025 Aug 22;20(8):e0330621. doi: 10.1371/journal.pone.0330621 (PMC12373185; doi:10.1371/journal.pone.0330621)
Supplement: S3 Fig — (PDF) [file pone.0330621.s004.pdf]

A

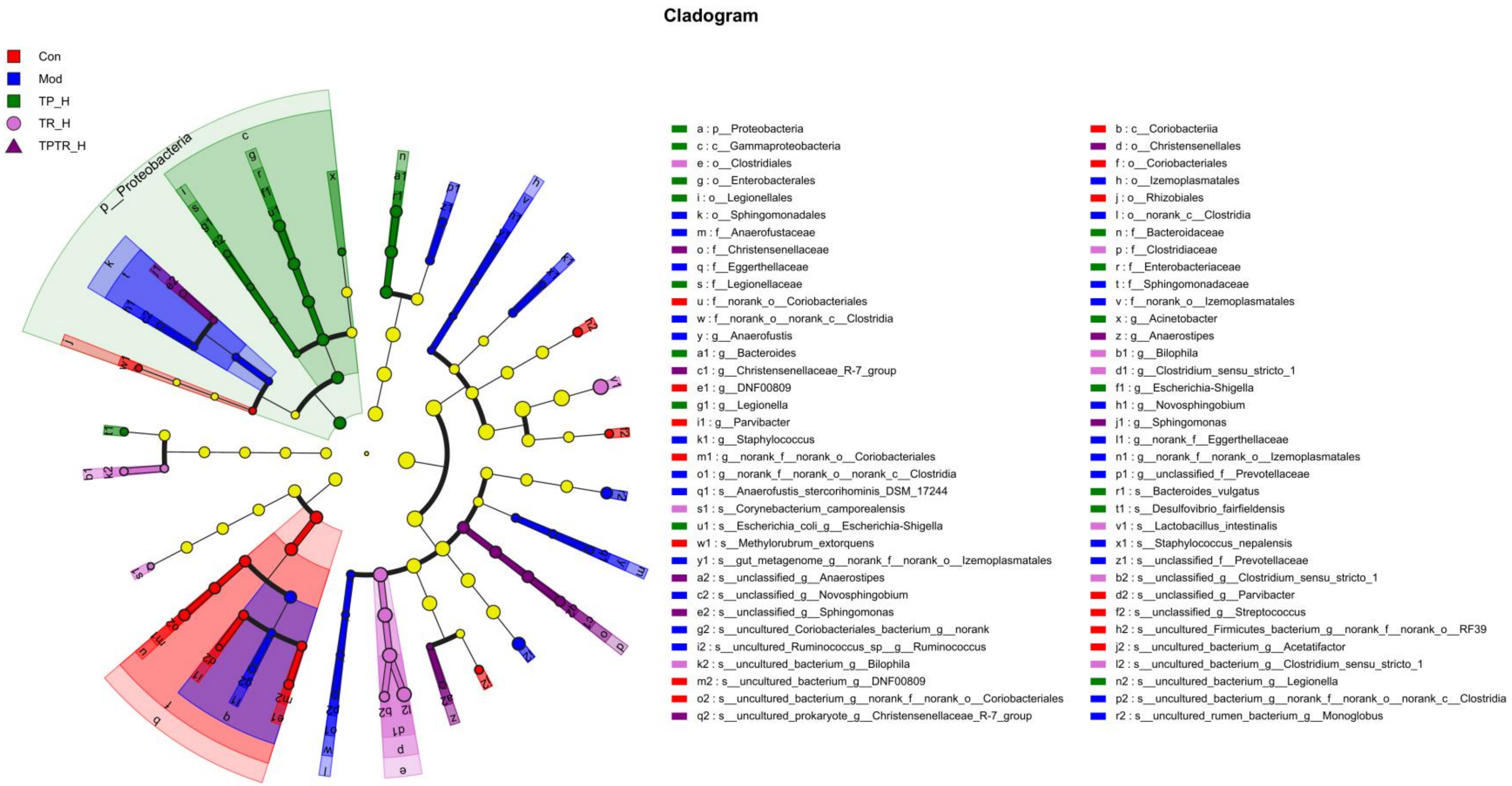
Cladogram
Con
Mod
TP_H
TR_H
TPTR_H
p__Proteobacteria
a : p__Proteobacteria
c : c__Gammaproteobacteria
e : o__Clostridiales
g : o__Enterobacterales
i : o__Legionellales
k : o__Sphingomonadales
m : f__Anaerofustaceae
o : f__Christensenellaceae
q : f__Eggerthellaceae
s : f__Legionellaceae
u : f__norank_o__Coriobacteriales
w : f__norank_o__norank_c__Clostridia
y : g__Anaerofustis
a1 : g__Bacteroides
c1 : g__Christensenellaceae_R-7_group
e1 : g__DNF00809
g1 : g__Legionella
i1 : g__Parvibacter
k1 : g__Staphylococcus
m1 : g__norank_f__norank_o__Coriobacteriales
o1 : g__norank_f__norank_o__norank_c__Clostridia
q1 : s__Anaerofustis_stercorihominis_DSM_17244
s1 : s__Corynebacterium_camporealensis
u1 : s__Escherichia_coli_g__Escherichia-Shigella
w1 : s__Methylorubrum_extorquens
y1 : s__gut_metagenome_g__norank_f__norank_o__Izemoplasmatales
a2 : s__unclassified_g__Anaerostipes
c2 : s__unclassified_g__Novosphingobium
e2 : s__unclassified_g__Sphingomonas
g2 : s__uncultured_Coriobacteriales_bacterium_g__norank
i2 : s__uncultured_Ruminococcus_sp__g__Ruminococcus
k2 : s__uncultured_bacterium_g__Bilophila
m2 : s__uncultured_bacterium_g__DNF00809
o2 : s__uncultured_bacterium_g__norank_f__norank_o__Coriobacteriales
q2 : s__uncultured_prokaryote_g__Christensenellaceae_R-7_group
b : c__Coriobacteriia
d : o__Christensenellales
f : o__Coriobacteriales
h : o__Izemoplasmatales
j : o__Rhizobiales
l : o__norank_c__Clostridia
n : f__Bacteroidaceae
p : f__Clostridiaceae
r : f__Enterobacteriaceae
t : f__Sphingomonadaceae
v : f__norank_o__Izemoplasmatales
x : g__Acinetobacter
z : g__Anaerostipes
b1 : g__Bilophila
d1 : g__Clostridium_sensu_stricto_1
f1 : g__Escherichia-Shigella
h1 : g__Novosphingobium
j1 : g__Sphingomonas
l1 : g__norank_f__Eggerthellaceae
n1 : g__norank_f__norank_o__Izemoplasmatales
p1 : g__unclassified_f__Prevotellaceae
r1 : s__Bacteroides_vulgatus
t1 : s__Desulfovibrio_fairfieldensis
v1 : s__Lactobacillus_intestinalis
x1 : s__Staphylococcus_nepalensis
z1 : s__unclassified_f__Prevotellaceae
b2 : s__unclassified_g__Clostridium_sensu_stricto_1
d2 : s__unclassified_g__Parvibacter
f2 : s__unclassified_g__Streptococcus
h2 : s__uncultured_Firmicutes_bacterium_g__norank_f__norank_o__RF39
j2 : s__uncultured_bacterium_g__Acetatifactor
l2 : s__uncultured_bacterium_g__Clostridium_sensu_stricto_1
n2 : s__uncultured_bacterium_g__Legionella
p2 : s__uncultured_bacterium_g__norank_f__norank_o__norank_c__Clostridia
r2 : s__uncultured_rumen_bacterium_g__Monoglobus

B

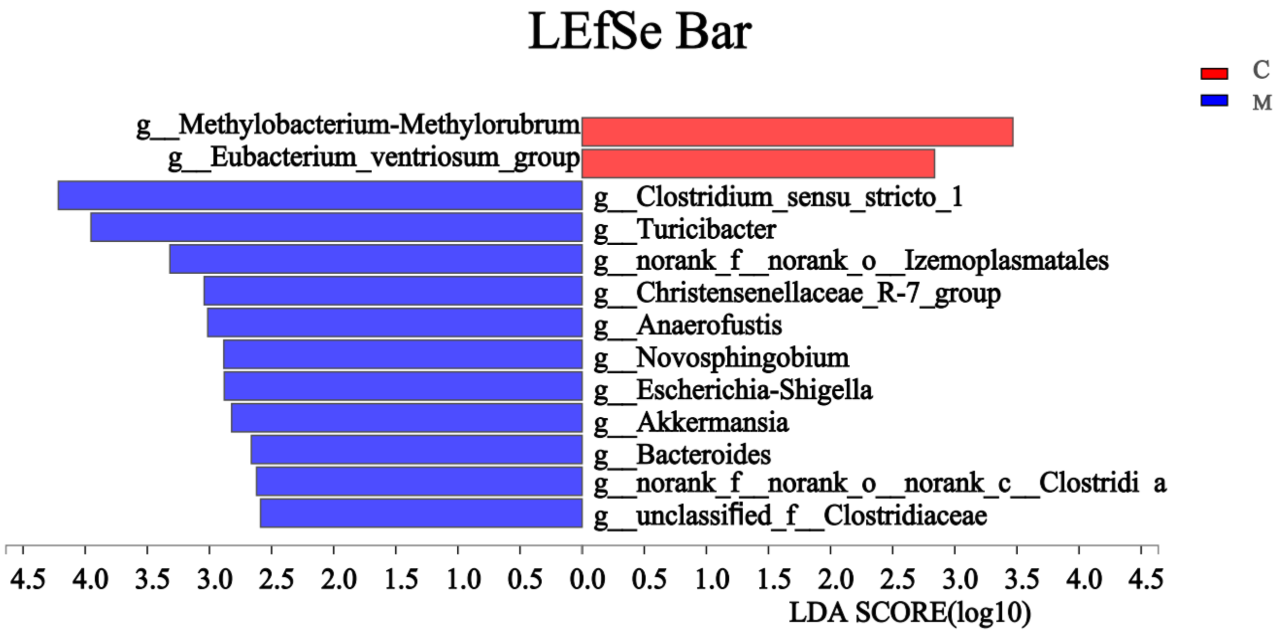

S3 Fig. Differential analysis of gut microbiota among different groups. (A) LEfSe plot cladogram of gut microbiota comparation among different groups. (B) LDA columnar graph.
