# Supplementary figures and images for: Investigating the Trichosanthis Pericarpium - Trichosanthis Radix herbal pair’s role in alleviating COPD through gut microbiota function, metabolomics analysis and cell validation experiment
Source: PLoS One. 2025 Aug 22;20(8):e0330621. doi: 10.1371/journal.pone.0330621 (PMC12373185; doi:10.1371/journal.pone.0330621)

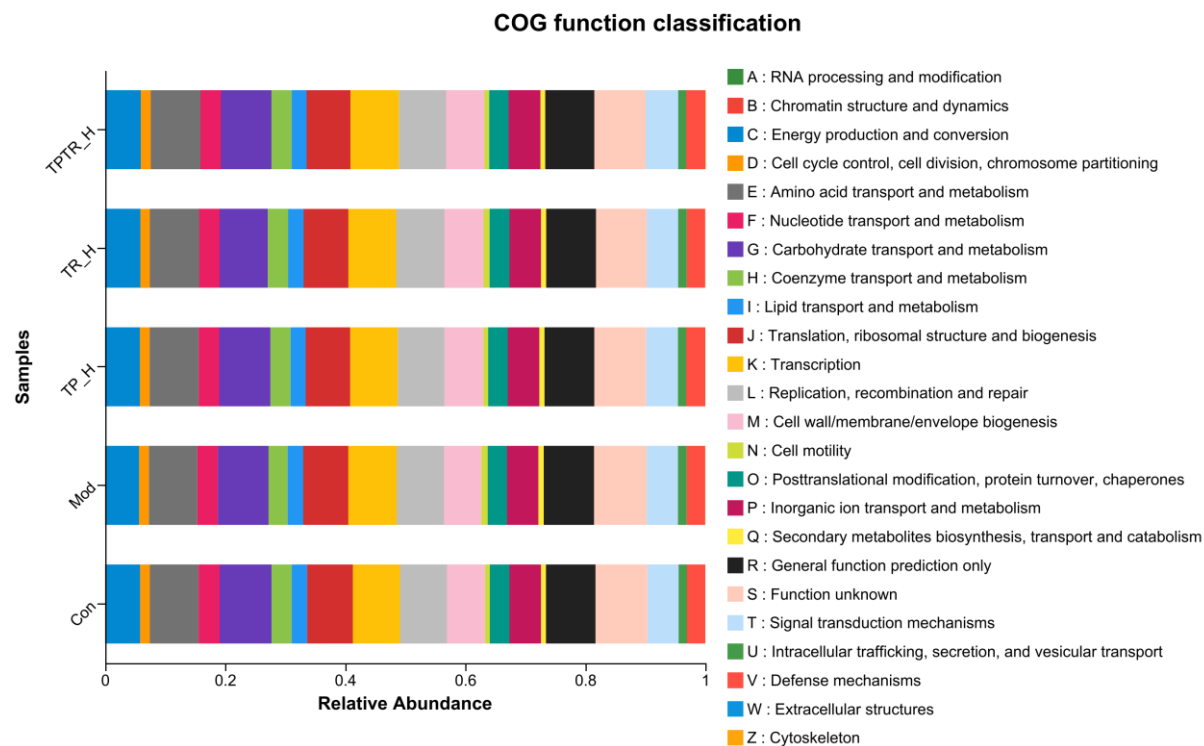

**S4 Fig.** COG function classification of gut microbiota in rats.

Supplement: S4 Fig — (PDF) [file pone.0330621.s005.pdf]

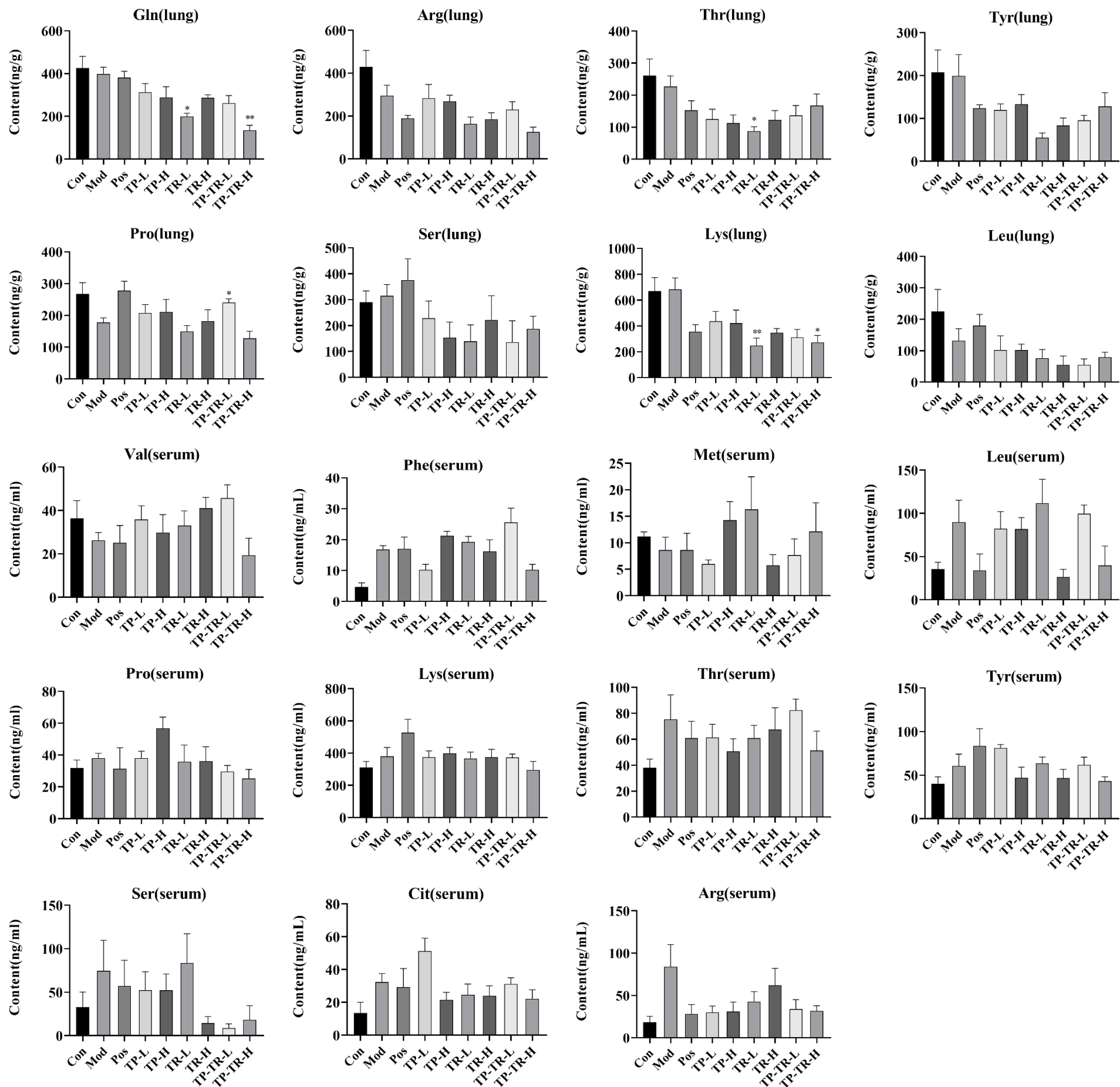

**S7 Fig.** Determination results of amino acid components in rat serum and lung tissue.

Supplement: S7 Fig — (PDF) [file pone.0330621.s008.pdf]

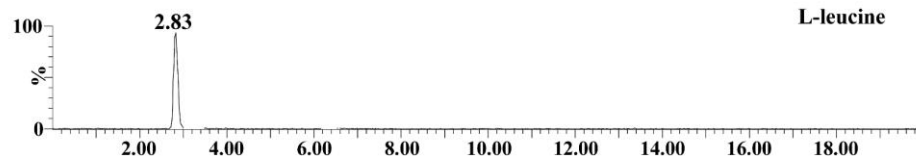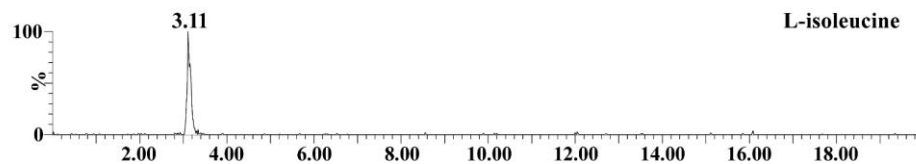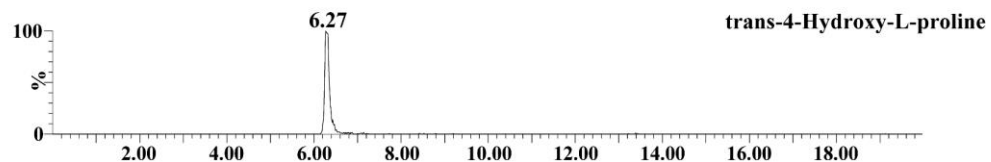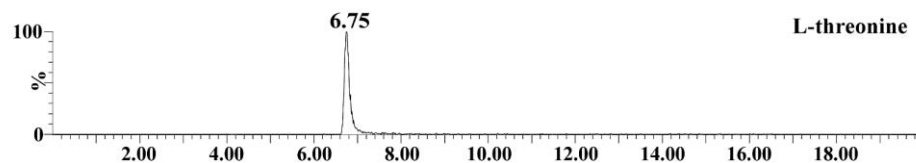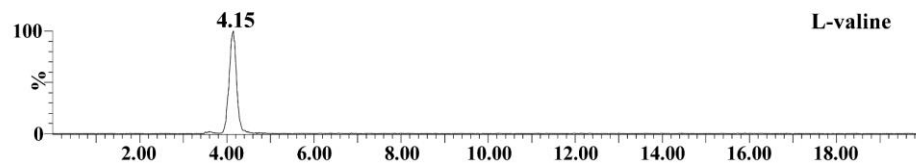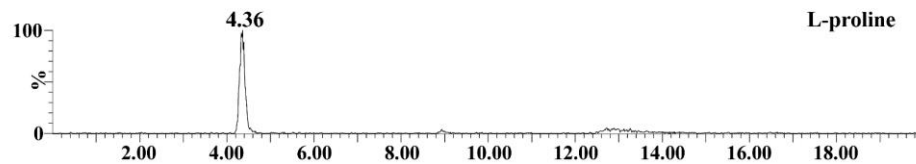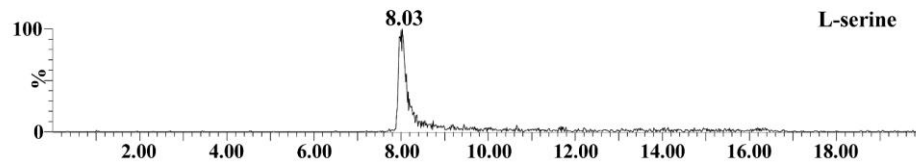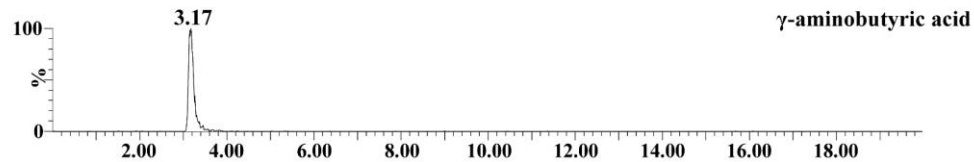

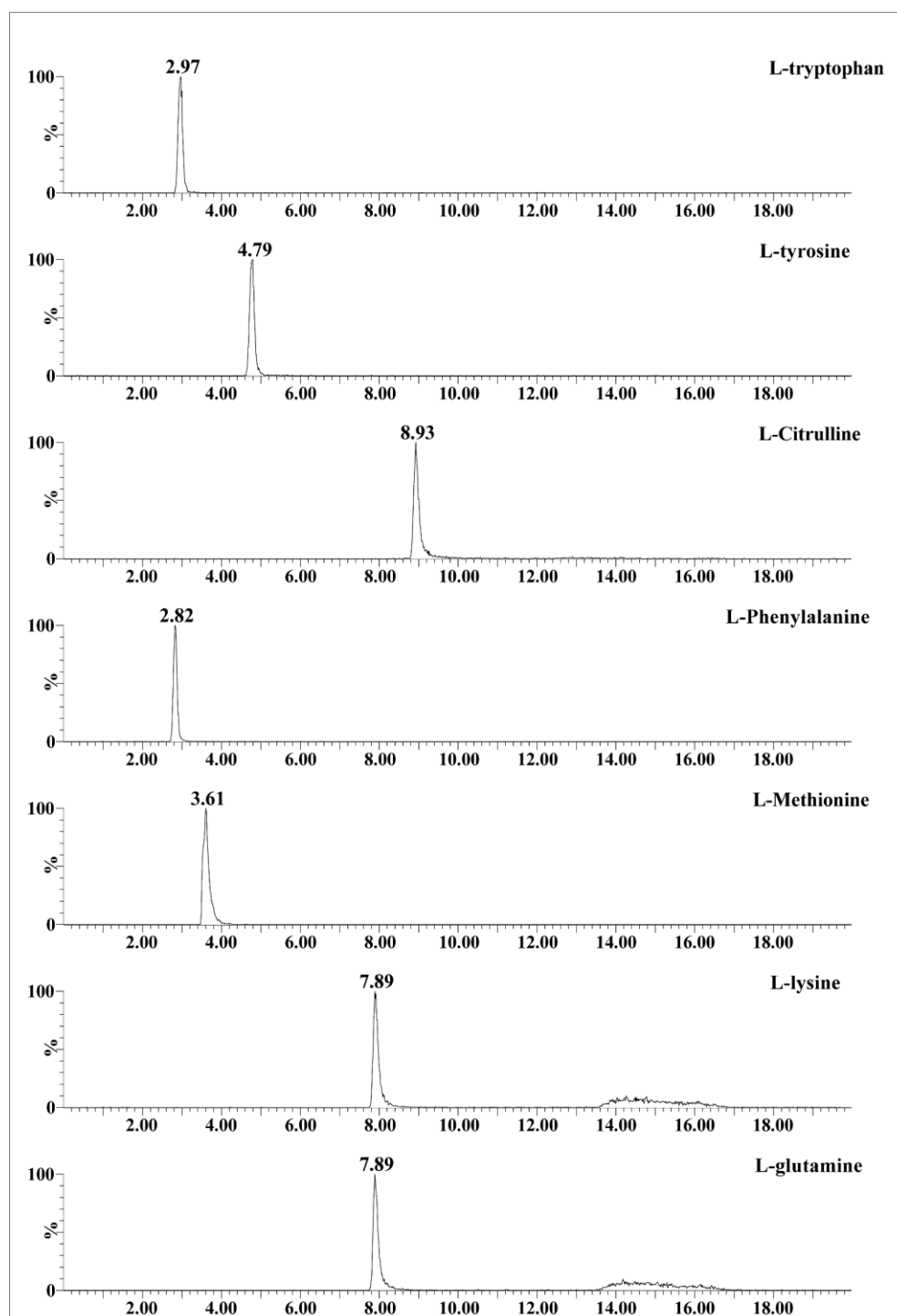

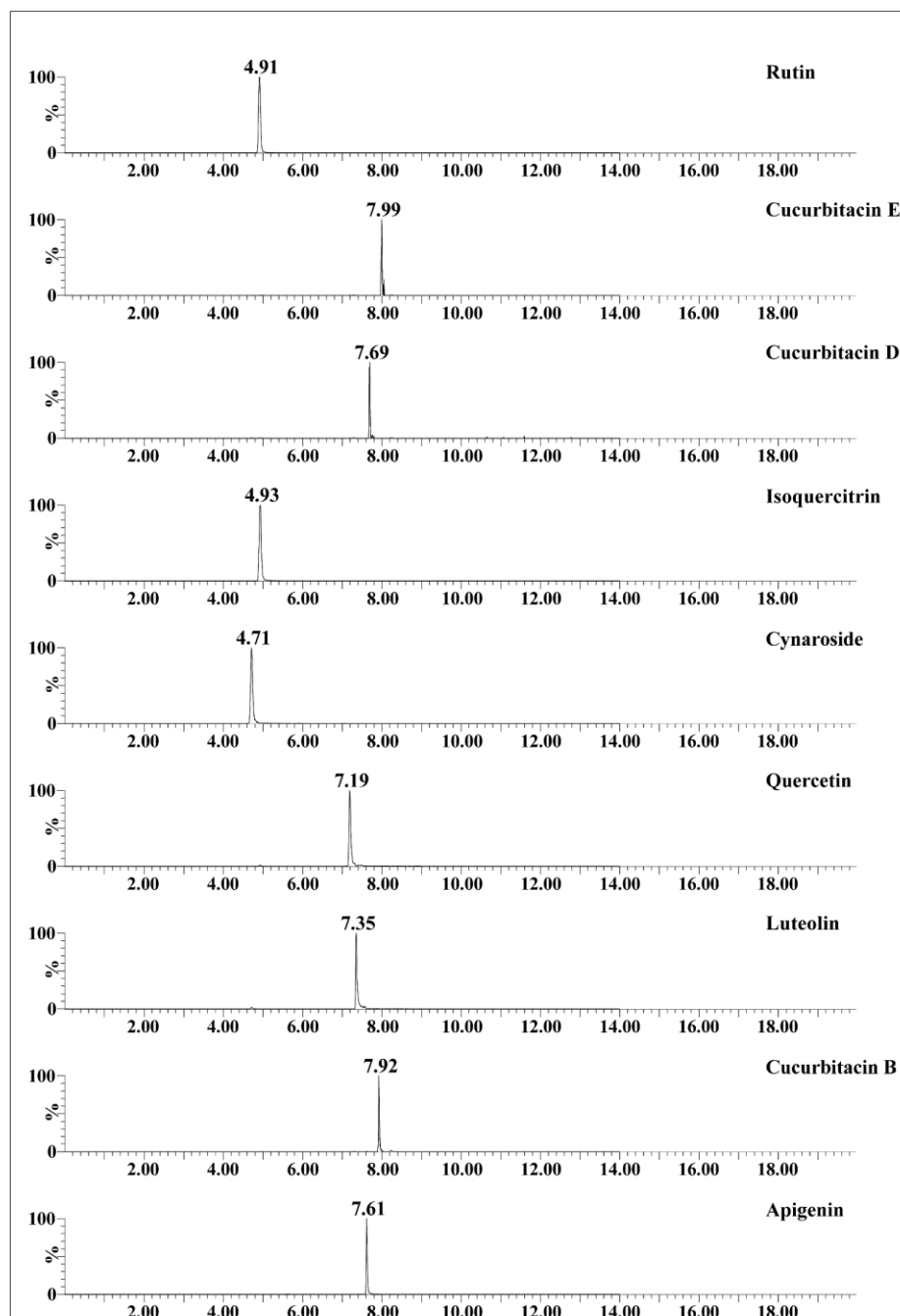

**S11 Fig.** The chemical profiles of flavonoids and amino acids in TP-TR.

Supplement: S11 Fig — (PDF) [file pone.0330621.s012.pdf]
