# Supplementary material for: Investigating the Trichosanthis Pericarpium - Trichosanthis Radix herbal pair’s role in alleviating COPD through gut microbiota function, metabolomics analysis and cell validation experiment
Source: PLoS One. 2025 Aug 22;20(8):e0330621. doi: 10.1371/journal.pone.0330621 (PMC12373185; doi:10.1371/journal.pone.0330621)
Supplement: S5 Fig — (PDF) [file pone.0330621.s006.pdf]

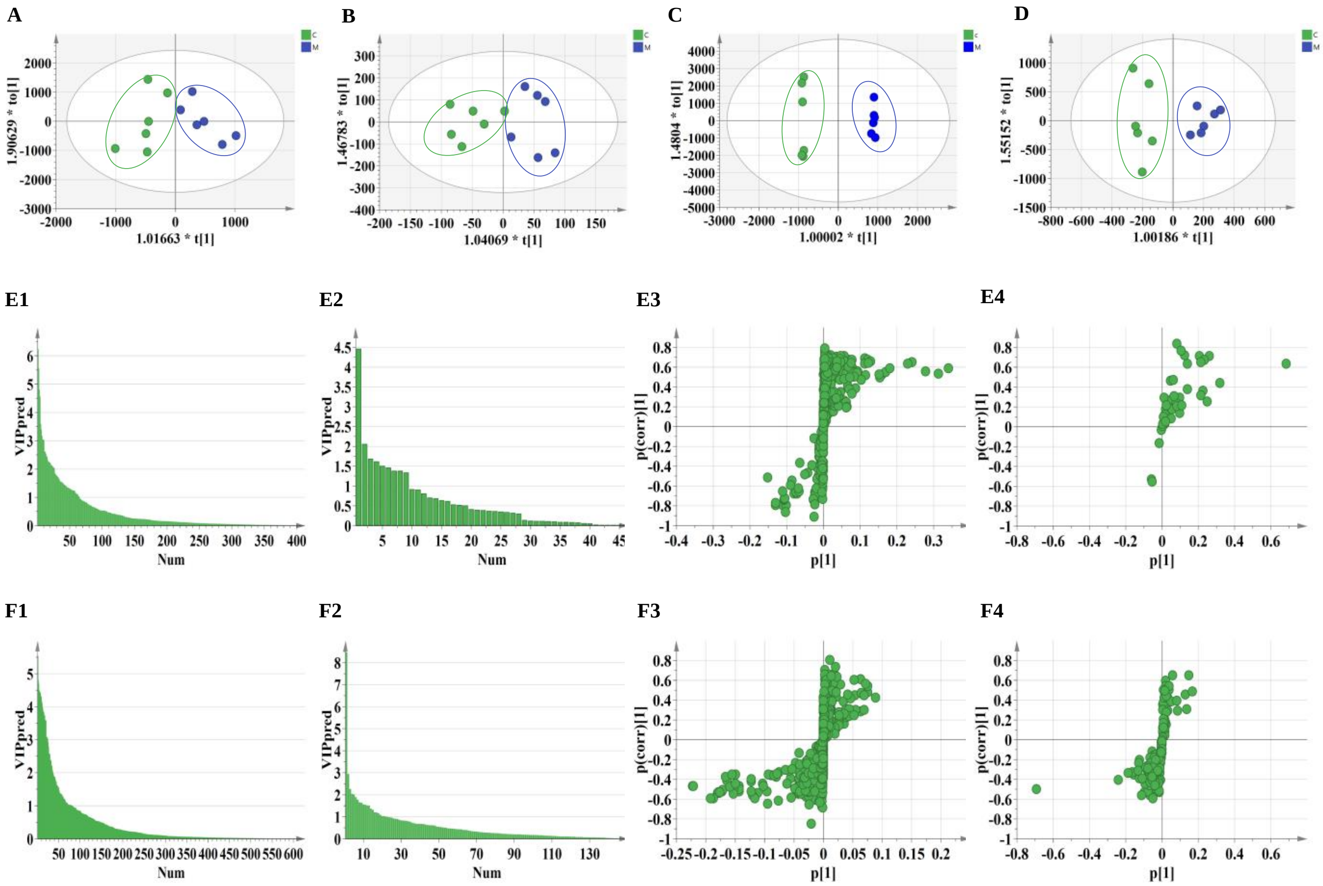

S5 Fig. OPLS-DA scoring map. (serum, A, ESI+; B, ESI-), (lung tissue, C, ESI+; D, ESI-); VIP and Loading S-plot by OPLA-DA analysis (E1, E3, serum positive ions; E2, E4, serum anion; F1, F3, positive ions of lung tissue; F2, F4, negative ions of lung tissue).
