# Supplementary material for: Investigating the Trichosanthis Pericarpium - Trichosanthis Radix herbal pair’s role in alleviating COPD through gut microbiota function, metabolomics analysis and cell validation experiment
Source: PLoS One. 2025 Aug 22;20(8):e0330621. doi: 10.1371/journal.pone.0330621 (PMC12373185; doi:10.1371/journal.pone.0330621)
Supplement: S6 Fig — (PDF) [file pone.0330621.s007.pdf]

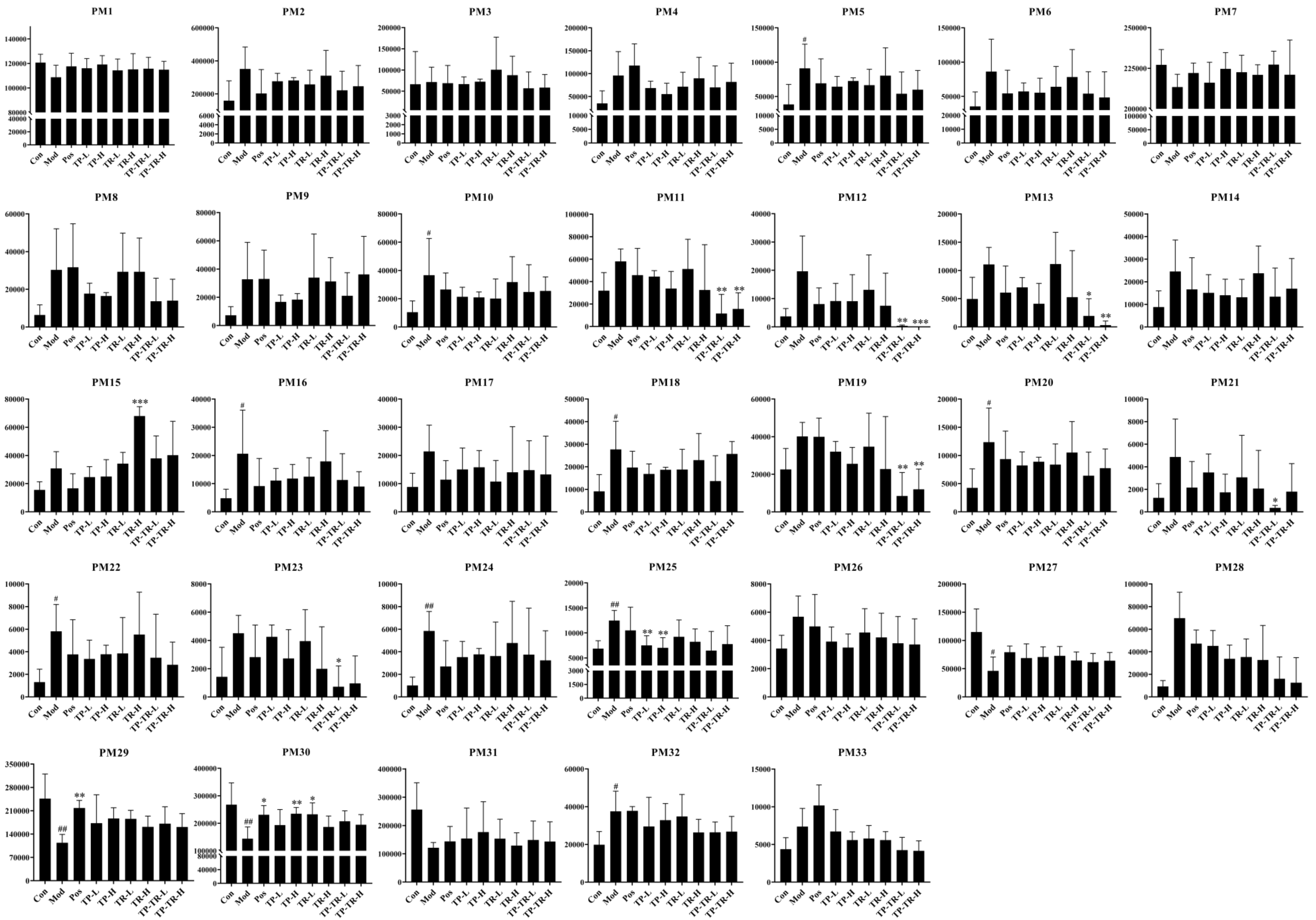

S6 Fig. Relative peak areas of potential markers in serum and lung tissue in positive and negative ion mode.
