# Supplementary material for: Investigating the Trichosanthis Pericarpium - Trichosanthis Radix herbal pair’s role in alleviating COPD through gut microbiota function, metabolomics analysis and cell validation experiment
Source: PLoS One. 2025 Aug 22;20(8):e0330621. doi: 10.1371/journal.pone.0330621 (PMC12373185; doi:10.1371/journal.pone.0330621)
Supplement: S10 Fig — (PDF) [file pone.0330621.s011.pdf]

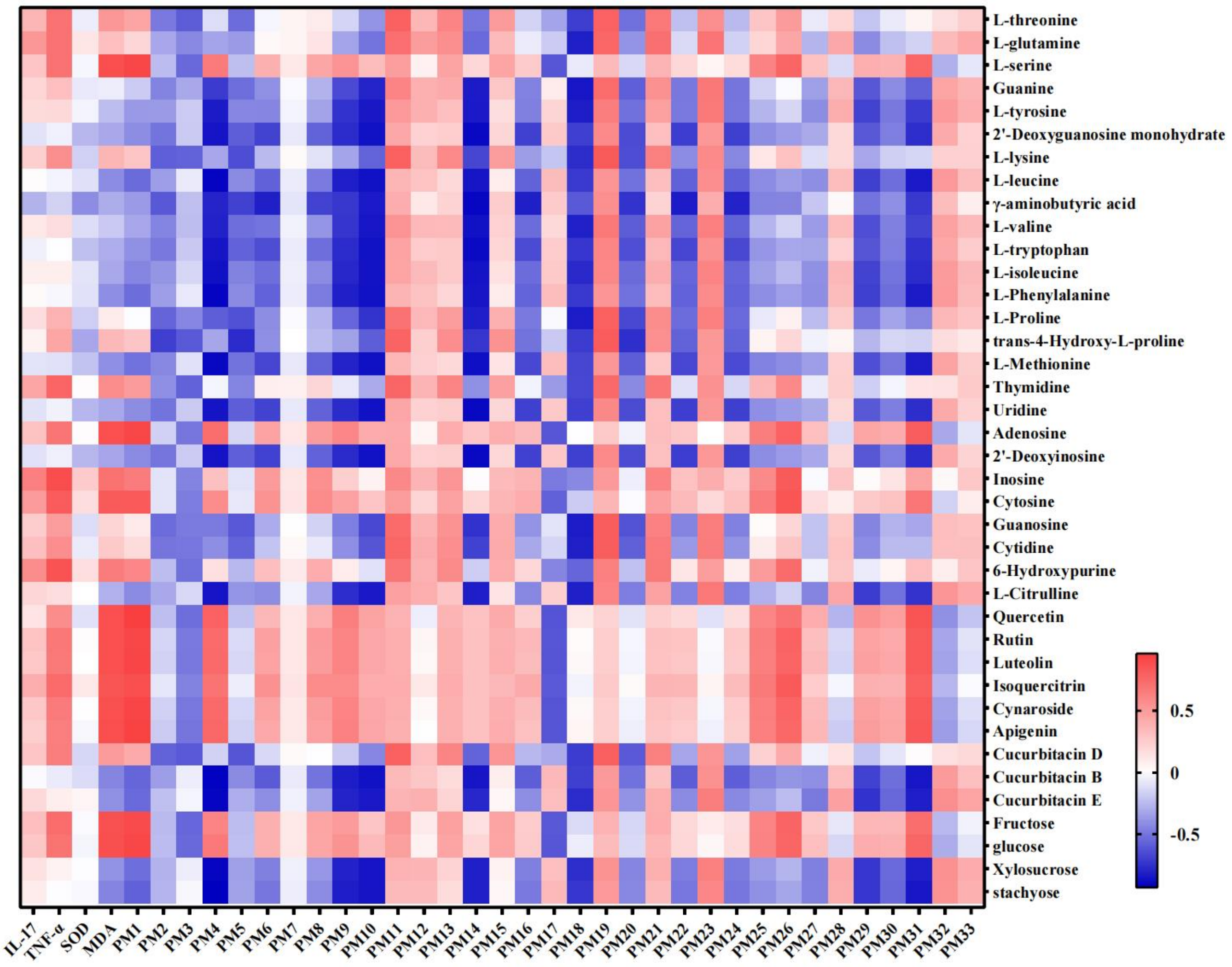

S10 Fig. Heat map of correlation coefficient between effect index, differential metabolites and component measurement index of Trichosanthis Pericarpium - Trichosanthis Radix herbal pair used to intervene COPD rats.
